# Whole proteome identification of plant candidate G-protein coupled receptors in *Arabidopsis*, rice, and poplar: computational prediction and *in-vivo *protein coupling

**DOI:** 10.1186/gb-2008-9-7-r120

**Published:** 2008-07-31

**Authors:** Timothy E Gookin, Junhyong Kim, Sarah M Assmann

**Affiliations:** 1 Department of Biology, The Pennsylvania State University, Mueller Laboratory, University Park, PA 16802, USA; 2 Department of Biology and Penn Genome Frontiers Institute, University of Pennsylvania, South University Avenue, Philadelphia, PA 19104, USA

## Abstract

**Background:**

The classic paradigm of heterotrimeric G-protein signaling describes a heptahelical, membrane-spanning G-protein coupled receptor that physically interacts with an intracellular Gα subunit of the G-protein heterotrimer to transduce signals. G-protein coupled receptors comprise the largest protein superfamily in metazoa and are physiologically important as they sense highly diverse stimuli and play key roles in human disease. The heterotrimeric G-protein signaling mechanism is conserved across metazoa, and also readily identifiable in plants, but the low sequence conservation of G-protein coupled receptors hampers the identification of novel ones. Using diverse computational methods, we performed whole-proteome analyses of the three dominant model plant species, the herbaceous dicot *Arabidopsis thaliana *(mouse-eared cress), the monocot *Oryza sativa *(rice), and the woody dicot *Populus trichocarpa *(poplar), to identify plant protein sequences most likely to be GPCRs.

**Results:**

Our stringent bioinformatic pipeline allowed the high confidence identification of candidate G-protein coupled receptors within the *Arabidopsis*, *Oryza*, and *Populus *proteomes. We extended these computational results through actual wet-bench experiments where we tested over half of our highest ranking *Arabidopsis *candidate G-protein coupled receptors for the ability to physically couple with GPA1, the sole Gα in *Arabidopsis*. We found that seven out of eight tested candidate G-protein coupled receptors do in fact interact with GPA1. We show through G-protein coupled receptor classification and molecular evolutionary analyses that both individual G-protein coupled receptor candidates and candidate G-protein coupled receptor families are conserved across plant species and that, in some cases, this conservation extends to metazoans.

**Conclusion:**

Our computational and wet-bench results provide the first step toward understanding the diversity, conservation, and functional roles of plant candidate G-protein coupled receptors.

## Abstract 2

Computational prediction and *in vivo* protein coupling experiments identify candidate plant G-protein coupled receptors in *Arabidopsis*, rice and poplar.

## Background

The ability to sense the environment and respond appropriately is a crucial factor for organism survival. One of the primary sensing mechanisms used by metazoans involves G-protein coupled receptor (GPCR) signaling cascades. These cascades are composed of, at the most simplistic level, a plasma membrane localized stimulus-sensing GPCR that transduces the extracellular signal to an intracellular heterotrimeric G-protein complex, thereby activating downstream signaling cascades. Because GPCR sequence conservation even within a single GPCR family of an organism can be lower than 25% [1], GPCRs are identified not by sequence homology but rather by their ability to couple with an intracellular heterotrimeric G-protein α subunit and by their two-dimensional topology, which classically consists of an extracellular amino terminus, seven membrane spanning domains connected by three intracellular and three extracellular loops, and an intracellularly located carboxy-terminal tail.

Signaling from the exterior of the cell is initiated when the GPCR becomes activated by ligand binding, stimulating an exchange of guanosine diphosphate for guanosine triphosphate on the Gα subunit, and a subsequent dissociation of the heterotrimer into Gα and a βγ subunit dimer. Gα and the βγ dimer then proceed to initiate downstream signaling cascades [2,3].

GPCRs comprise the largest class of transmembrane signaling molecules present in metazoan organisms and have been shown to recognize ligands and effectors such as photons, ions, nucleotides, amino acids, peptides, glycoproteins, hormones and lipids [4,5]. Although GPCRs appear to be strictly limited to the eukaryota, they are ubiquitous and have been cloned from a wide range of evolutionarily distant organisms, including yeast [6], coral [7], nematodes [8], arthropods [9], human [10], and even from the preserved DNA of the woolly mammoth [11]. GPCRs play central roles in processes as diverse as yeast mating and insect taste perception [12], and in mammals, GPCR signaling plays critical roles in development and metabolism. Aberrant mammalian GPCR activity has been directly linked to such maladies as blindness, asthma, heart disease, obesity, and cancer [13,14].

Whole genome sequencing efforts have shown that heterotrimeric G-protein signaling can be highly complex. The human proteome is known to contain 23 Gα, 5 Gβ, and 12 Gγ subunits [15], leading to over 1,300 theoretical heterotrimeric complexes. Factoring in the over 850 predicted human GPCRs [16], many of which are known to homo- and heterodimerize [17], the number of potential signaling pathways becomes enormous. In sharp contrast, the number of known heterotrimeric signaling complex components in plants is dramatically less. The fully sequenced model plant *Arabidopsis thaliana *has only one canonical Gα subunit (GPA1), one Gβ subunit (AGB1), and two identified Gγ subunits (AGG1 and AGG2) [18,19]. *Arabidopsis *also has a single regulator of G-protein signaling (RGS) protein (RGS1), which has been shown to directly accelerate the intrinsic guanosine triphosphatase activity of Gα [20]. Interestingly, RGS1 contains a heptahelical domain as well as an RGS box domain, and might also function as a receptor or co-receptor [21]. For the past decade there has been only one putative GPCR (GCR1) identified and experimentally investigated in *Arabidopsis *[22-25]. Recently, a new GPCR, GCR2, has been reported in *Arabidopsis *[26], although this protein sequence does not appear to have the canonical seven transmembrane (TM) topology of known GPCRs and some discrepancies exist regarding its purported plant hormone signaling function [27]. Thus, the question that arises, and which is the focus of the present study, is whether the *Arabidopsis *genome is as depauperate of GPCRs as it is of heterotrimeric G-protein subunits, or whether additional *Arabidopsis *GPCRs exist that have not yet been identified. In other words, given that *Arabidopsis *has only one canonical Gα subunit and one canonical Gβ subunit [28,29], and only two identified Gγ subunits [30,31], is it reasonable that GCR1, and potentially RGS1, are the only candidate GPCRs in *Arabidopsis*, or are there other as yet undiscovered candidate GPCRs? The large number of plant responses that are affected upon genetic knockout of *GPA1*, *AGB1*, *AGG1*, or *AGG2 *[32,33] suggests that the latter hypothesis may prove true.

The great physiological importance of GPCRs, combined with the ever-increasing availability of nucleic acid sequence data, has prompted the development and use of bioinformatic tools to predict and identify new GPCRs. Using both functionally characterized GPCRs and their predicted sequence homologs as a starting point, new predicted GPCRs have been identified and shown to be plentiful in a broad range of organisms from slime molds to humans [16]. Analyses based on sequence conservation are useful for identifying GPCRs that are highly similar to known GPCRs, but the low sequence conservation within the GPCR superfamily, and even within each GPCR family, limits this approach. To circumvent this problem, more comprehensive bioinformatic methods have been developed to identify and characterize potential GPCRs.

More than ten bioinformatic programs designed to identify transmembrane domains are publicly available, and programs such as TMHMM2 [34] and HMMTOP2 [35,36], and Phobius [37,38] can be used to identify sequences with the classic 7TM domain topology of GPCRs. In a comparative study, Cuthbertson *et al*. [39] found TMHMM2 and HMMTOP2 to consistently perform better than other programs, and Phobius was reported to perform comparably [38]. To attain greater accuracy in the number of predicted TMs, signal peptide prediction programs such as Phobius and Signal-P [40] can be used in conjunction with dedicated TM prediction programs, since TM domain predictors alone have a tendency to mistakenly predict signal peptides as amino-terminal transmembrane domains [41-43].

At the level of directly predicting a sequence as a GPCR there are only a few prediction methods available, and the diversity in their approach is an indicator of the difficulty of this task. The quasi-periodic feature classifier (QFC) developed by Kim *et al*. [44] maps statistical values derived from protein sequence attributes into an n-dimensional feature space and classifies the query sequence as either a GPCR or a non-GPCR through the use of a discriminate function. The QFC relies on four parameters for classification: amino acid usage index; log of the average periodicity of the hydrophobicity function; log of average periodicity of the polarity scale; and variance of the first derivative of the polarity scale. Notably, the QFC has been used successfully to identify *Drosophila *odorant and gustatory receptors [45,46] and *Anopheles *odorant receptors [47].

The GPCRHMM [48] prediction method is based on variances in amino acid composition and topological segment lengths between GPCR families. While not explicitly predicting a 7TM topology, GPCRHMM describes the typical 7TM topology by creating different hidden state compartments to model each of the three extracellular segments, the three intracellular segments, and the seven transmembrane segments that connect them. The amino and carboxyl termini are additionally broken into two compartments (close to the membrane and globular) and the distal amino-terminal compartment also includes a signal peptide model. GPCRHMM also includes a secondary filter that takes sequences passing the global prediction model and re-analyzes the central core 7TM region of the query using only the corresponding local compartment models in order to reduce the number of false positives arising from amino acid composition bias derived from long amino and carboxyl termini.

Recently, Moriyama *et al*. [49] combined the alignment free methods of discriminant function analyses, support vector machines, and partial least squares regression (LDA, QDA, KNN, SVM-AA, SVM-di, and PLS-ACC) to identify a preliminary list of 652 *Arabidopsis *candidate 7TM receptors. This initial list was reduced by filtering with HMMTOP2 [36] to tentatively identify 394 putative 7TM receptor proteins (7TMpRs) with 5-10 predicted TM domains. A subsequent requirement of exactly seven predicted TM domains and an extracellular amino terminus identified 54 non-redundant proteins as 7TMpRs. This prediction method has not been challenged in biological experiments in order to determine if the predicted GPCRs actually couple to a Gα subunit.

In our work we use a combination of direct GPCR prediction methods, multiple TM domain prediction analyses, and signal peptide prediction to identify and rank candidate GPCRs in the *Arabidopsis *proteome. Once potential candidate GPCRs have been identified in a proteome, it is possible to classify them using software such as the four level classifier GPCRsIdentifier [50], which classifies GPCRs as belonging to GPCR superfamily, family, sub-family, and sub-family types based on amino acid composition and dipeptide frequencies. Beyond classification, candidate GPCRs can be characterized using coupling specificity prediction software such as Pred-Couple 2 [51], which predicts the type of Gα subunit with which the candidate GPCR should physically interact. We further characterize our candidate GPCRs by using GPCRsIdentifier to classify our candidate plant GPCRs and Pred-Couple 2 to predict their coupling specificity. We also show evidence for evolutionary conservation of our identified candidate GPCRs using the fully sequenced genomes of rice (*Oryza sativa*) and poplar *(Populus trichocarpa*), and search the Pfam database [52] to investigate domain similarities. Most importantly, we also provide positive results from *in vivo *protein-protein coupling assays between some of our highest ranking *Arabidopsis *candidate GPCRs and the sole Gα subunit in *Arabidopsis*, thus confirming the efficacy of our bioinformatic scheme for identifying novel, divergent GPCRs.

## Results

### Identification of candidate GPCRs *in Arabidopsis*

**Figure 1:**
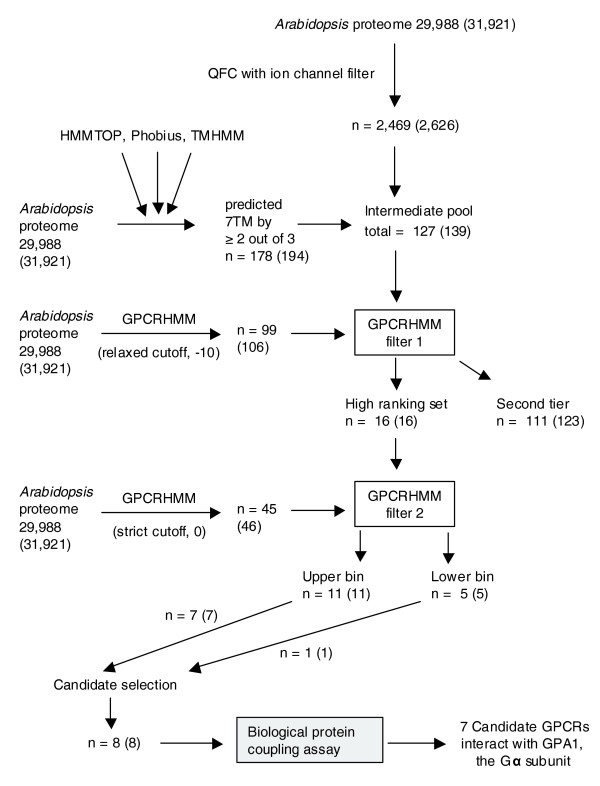
Flowchart detailing our *A. thaliana *candidate GPCR identification and *in vivo *analysis scheme. Numbers in parentheses include redundant protein sequences. A complete list of splice variants and redundant proteins for the *Arabidopsis *proteome is supplied in Additional data file 12.

Due to the low sequence similarity of GPCRs, alternative methods beyond BLAST are required to identify novel GPCRs. Because the QFC algorithm was reported to have an approximately 98% success rate in classifying GPCRs from non-GPCRs [44], and GPCRs are classically described by their 7TM topology, our criterion to identify a protein sequence as a candidate GPCR comprises the requirements of direct prediction as a GPCR by the QFC algorithm and the presence of exactly seven TM domains as predicted by at least two of the three TM prediction programs used (TMHMM2, HMMMTOP2, and Phobius) after correction for signal peptide misprediction (Figure 1).

**Figure 2:**
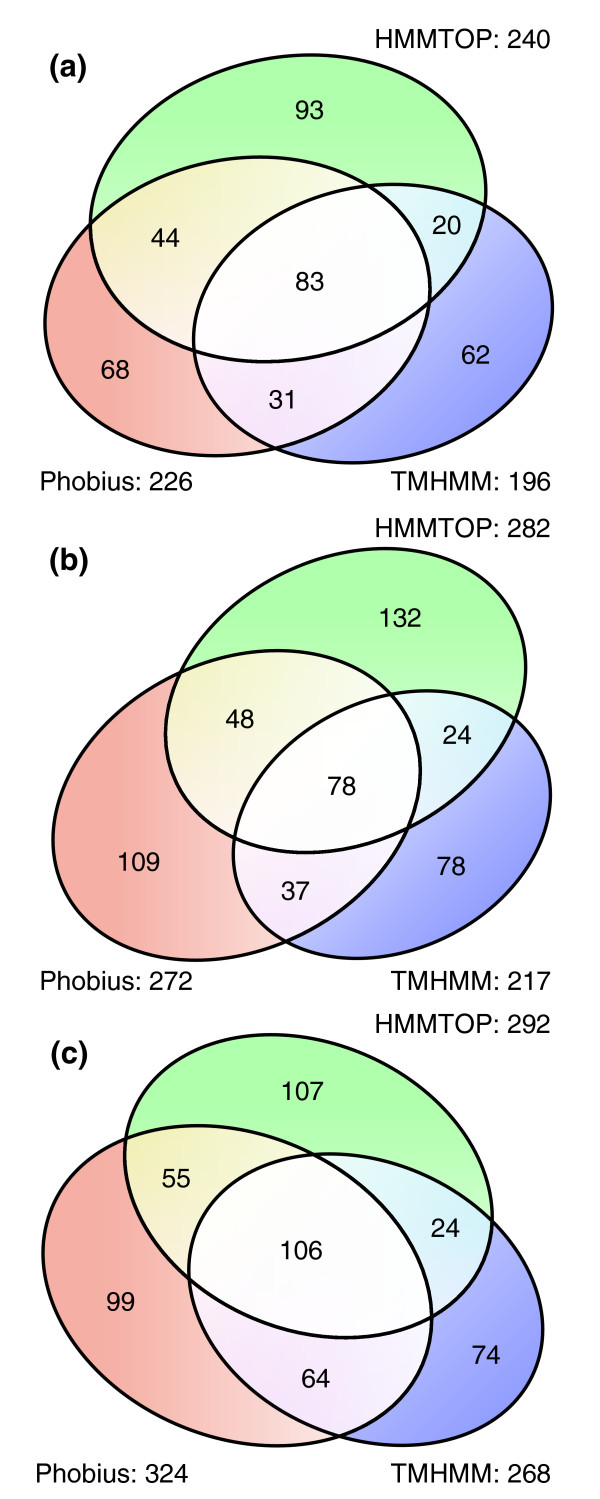
Proportional Venn diagrams detailing the number of predicted and co-predicted 7TM protein sequences in the non-redundant **(a) ***Arabidopsis*, **(b) ***Oryza*, and **(c) ***Populus *proteomes. Signal peptides were removed *in silico *prior to topology analyses.

We identified 2,469 *Arabidopsis *proteins that satisfied the QFC requirement (Figure 1). To predict proteins containing seven TM domains, we performed whole proteome analyses with the dedicated TM prediction programs TMHMM2 and HMMTOP2, and the signal peptide/TM domain co-prediction program Phobius. The mature proteins of sequences with signal peptides detected by Phobius were subsequently re-analyzed by TMHMM2 and HMMTOP2 (Figure 2a). A total of 401 non-redundant protein sequences were predicted to have seven TM domains by at least one of the three programs and 83 were predicted to have seven TM domains by all three. We identified 178 proteins that satisfied our '2/3 predictions' rule for the presence of exactly seven TM domains (Figure 2a). The intersecting set of these 178 proteins with the 2,469 proteins identified by QFC analysis contains 127 candidate GPCRs, which we call the 'intermediate pool'; of these, 71 are predicted to have exactly seven TM domains by all three TM domain predictors (Figure 1; Additional data file 1).

From this intermediate pool of 127 proteins, we designated a sequence as a high ranking candidate GPCR if it also satisfied the criterion of prediction as a GPCR by GPCRHMM using the relaxed global threshold of -10. Because GPCRHMM appears to have high specificity for selecting GPCRs, and it has been reported that reducing the GPCRHMM global cutoff threshold to as low as -53 still allows GPCRHMM to function with a false positive rate of only approximately 1% when analyzing data sets composed of proteins containing 6-8TMs [48], we chose to use the relaxed global threshold of -10 in order to select more divergent GPCR candidates while still minimizing the number of false positives. Whole *Arabidopsis *proteome analysis by GPCRHMM using this threshold identified a non-redundant set of 99 sequences (Figure 1). Of these 99 sequences, 16 also satisfied the prediction criteria from our QFC and 7TM analyses; thus, we designated these 16 as 'high ranking' candidate GPCRs (Figure 1; Table 1), while the remaining 111 proteins from the intermediate pool were designated as second tier GPCR candidates. Further filtering of the 16 sequences through the use of a stricter global threshold in combination with a local GPCRHMM filter, namely a GPCRHMM global filter threshold level of 0 and a positive GPCRHMM local score, identified 11 of the 16 candidates as belonging to an upper bin within the set of high ranking candidate GPCRs (Table 1).

**Table 1:** Characterization of our high ranking *Arabidopsis *candidate G-protein coupled receptors

Locus	ID	QFC	GPCRHMM	TMHMM	HMMTOP	Phobius	Pcut-T	Pcut-H
Upper bin								
**At1g48270.1**^‡^	GCR1	*	*	7 (out)	7 (out)	7 (out)		
**At3g26090.1**^†^	RGS1	*	*	7 (out)	7 (out)	7 (out)		
**At1g57680.1**	**Cand1**	*	*	7 (out)	7 (out)	7 (out)		
**At3g05010.1**	**Cand2**	*	*	7 (in)	7 (out)	7 (out)		
**At3g59090.1**	**Cand3**	*	*	7 (out)	7 (out)	7 (out)		
At3g59090.2	**Cand4**	*	*	7 (out)	7 (out)	7 (out)		
**At3g59090.3**	**Cand5**	*	*	7 (out)	7 (out)	7 (out)		
*At4g21790.1*	TOM1	*	*	7 (out)	7 (out)	8 (in)		
At5g02630.1	Cand6	*	*	6 (out)	8 (out)	7 (out)	7 (in)	7 (in)
**At5g18520.1**	**Cand7**	*	*	8 (in)	8 (in)	7 (out)	7 (out)	7 (out)
**At5g27210.1**	**Cand8**	*	*	7 (in)	7 (out)	7 (out)		
								
Lower bin								
At2g02180.1	TOM3	*	-0.47	6 (in)	7 (out)	7 (out)		
**At4g30850.1**	**HHP2**	*	-4.83	7 (in)	7 (in)	8 (out)		
At5g26740.1	Cand9	*	-7.95	7 (out)	7 (out)	7 (out)		
At1g14530.1	THH1	*	-8.95	6 (in)	7 (out)	7 (out)		
At3g05940.1	Cand10	*	-9.89	7 (out)	7 (out)	7 (out)		

Protein sequences predicted to be GPCRs by QFC and GPCRHMM are indicated by an asterisk and the GPCRHMM global score is provided for sequences we identify as GPCRs using a relaxed cutoff threshold score of -10. The predicted number of transmembrane domains and the intracellular (in) or extracellular (out) localization of the amino terminus are shown. Pcut-T and Pcut-H describe topology predictions of the mature proteins by TMHMM and HMMTOP, respectively, after *in silico *cleavage at the signal peptide cleavage site predicted by Phobius. Candidates shown to interact with GPA1 *in vivo *using the split-ubiquitin system are identified in bold while the sole negative result from that assay is shown in italics. ^‡^GCR1 interaction with GPA1 in the split-ubiquitin system was previously described by Pandey and Assmann [60]. ^†^RGS1 interaction with GPA1 in the split-ubiquitin system was previously described by Chen *et al*. [20]; the RGS1 sequence was truncated at the upstream border of the RGS box prior to analysis by GPCRHMM.

Twelve of the sixteen high ranking candidate GPCR sequences were predicted to have seven TM domains by all three methods (Table 1), with ten of the consensus 7TM proteins found within the eleven member upper bin. Two of the upper bin consensus 7TM predictions (Cand6, At5g02630.1; Cand7, At5g18520.1) are only apparent after removal of the signal peptide (Table 1).

### Empirical testing of *Arabidopsis *candidate GPCR Gα-coupling ability

Although the identification of candidate GPCRs by bioinformatic means is informative, the validity of the predictions can only be determined empirically. One obvious criterion that GPCR proteins should logically satisfy is that they should physically interact with a G-protein α subunit. As wet-bench evaluation of such protein-protein interactions is not a trivial task, we chose half of our *Arabidopsis *high ranking candidate GPCRs for *in vivo *analysis, and did so using additional information beyond our initial criteria of direct GPCR prediction and TM domain analysis.

Candidates Cand2 and Cand8 were chosen based on their limited similarity to GPR175, a mammalian GPCR. Heptahelical protein 2 (HHP2) was selected for analysis since the HHP family shows similarity to the atypical GPCRs of the human adiponectin receptor and membrane progestin receptor family [53]. The Tobamovirus replication protein TOM1 sequence was selected for analysis since both TOM1 and TOM3 were shown to be essential for tobamovirus pathogenicity in *Arabidopsis *[54] and mammalian GPCRs are essential for HIV pathogenesis [55]. Two of the splice variant products encoded by the At3g59090 locus (Cand3 and Cand5) were chosen based on the fact that they differ primarily in their amino-terminal regions and both are annotated as being similar to TOM1. Our BLAST analyses show that Cand3 and Cand5 have only limited similarity to TOM1 or TOM3, with BLAST e-values ranging between e^-12 ^and e^-07 ^(data not shown).

A high proportion of GPCRs, especially class A GPCRs, are known to be intronless [56], and this information was used to select Cand1 and Cand7 instead of other candidates that, like Cand1 and Cand7, are also annotated only as expressed proteins. Additional support for selecting Cand7 came from domain prediction analyses using the conserved domain database at NCBI, which indicated that Cand7 has a Lung 7TM receptor domain with a query e-value of 3.1e^-35^.

**Figure 3:**
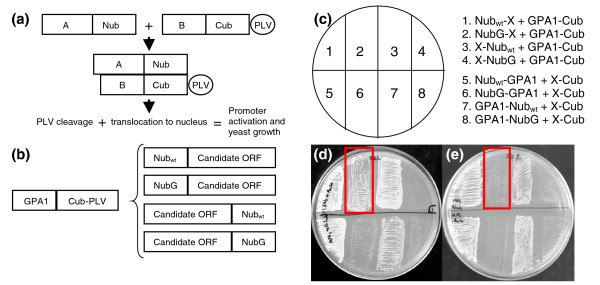
Experimental organization and two representative results for GPA1-candidate GPCR interaction assessed by the split-ubiquitin system. **(a) **Schematic showing a simplified outline of the split-ubiquitin system assay: protein A is fused to the amino-terminal half of ubiquitin as an amino- or carboxy-terminal fusion (only the amino-terminal fusion orientation is shown here); protein B is fused to the carboxy-terminal half of ubiquitin, which in turn has a fused artificial transcription factor (PLV). Interaction of protein A with protein B brings the two halves of ubiquitin into close proximity and a functional ubiquitin molecule is restored. Ubiquitin specific proteases cleave off PLV, which translocates to the nucleus and activates transcription of target genes allowing for yeast growth. **(b) **Cartoon detailing the control (Nub_wt_) and test (NubG) fusion protein construct orientations for sectors 1-4 in (c). **(c) **Schematic depicting the organization of the interaction assay plates in (d,e). The X represents the candidate GPCR open reading frame (ORF). Sectors 5-8 show the reciprocal assay. **(d,e) **Representative results for the ability of candidate GPCRs to interact with GPA1, the Gα subunit, on 1 mM methionine repression media. Diploid yeast containing GPA1 fusion constructs and either candidate Cand5 (d) or TOM1 (e) fusion constructs both grow on minimal media (not shown), but Cand5 specifically interacts with GPA1 and allows growth on the repression media (d, boxed sector) while TOM1 does not (e, boxed sector).

After choosing these candidates, we applied the split-ubiquitin system to test their ability to interact with GPA1, the sole canonical G-protein α subunit of *Arabidopsis*. The split-ubiquitin system variant of the yeast two hybrid assay is based on the ability of the amino-terminal (Nub_wt_) and carboxy-terminal (Cub) domains of ubiquitin to spontaneously reassemble and become a functionally recognized target for ubiquitin specific proteases, which cleave an artificial transcription factor, PLV, that is fused downstream of Cub (Figure 3a). PLV translocation to the nucleus and subsequent induction of reporter gene expression leads to functional complementation of auxotrophic yeast and positive interactions are easily visualized through yeast growth. Protein-protein interaction test assays are possible through the use of NubG, a mutant version of Nub_wt _that has reduced affinity for Cub; thus, a functional ubiquitin is reassembled only if the two test proteins (in our case, a candidate GPCR and GPA1) interact. Increased assay stringency is achieved by modulating test protein expression levels through the application of methionine, which downregulates the methionine repressible Met25 promoter that drives Cub fusion protein expression. In our split-ubiquitin system assays we separately fused the Nub_wt _and NubG domains to both the amino terminus and carboxyl terminus of the candidate GPCR and tested the ability of these fusion proteins to interact with the GPA1-Cub-PLV fusion protein (Figure 3b,c, sectors 1-4). Fusion with the Nub_wt _is a positive control that should always yield protein-protein interaction. Because the fusion of additional protein sequence can cause physiochemical changes in protein structure and loss of function, we also performed the reciprocal assay in which the Nub domains were fused to GPA1, and the candidate GPCR was fused to Cub-PLV (Figure 3c, sectors 5-8). The two reciprocal assays were performed on the same methionine supplemented media plate (Figure 3c). Since a lack of yeast growth indicates a lack of protein-protein interaction, all interaction assay cultures were simultaneously verified as capable of growing on minimal media alone (data not shown). All of the positive interactions, as determined by yeast growth due to complementation of the *his3 *mutation, were also accompanied by the expected color change of the diploid yeast due to complementation of the *ade2 *mutation (data not shown).

Figure 3d illustrates one positive result, while Figure 3e illustrates the sole negative result from our tests of candidate GPCRs. As shown in sector 2 of Figure 3d, the candidate GPCR Cand5 interacts with GPA1 as shown by the presence of yeast growth; however, this interaction does not occur when the Cand5 protein has a carboxy-terminal NubG or Cub fusion protein (Figure 3d, sectors 4, 6 and 8), consistent with the known importance of GPCR carboxyl termini in binding G-proteins as well as other GPCR interacting proteins [57]. As shown in Figure 3e, sectors 2, 4, 6 and 8 all lack yeast growth, demonstrating that this candidate GPCR, TOM1, does not interact with GPA1 regardless of the orientation of the fusion proteins.

Our complete results, summarized in Table 2, demonstrate that seven of the eight candidate GPCRs we tested indeed interact with GPA1. All of the positive control interactions using Nub_wt_-candidate fusion proteins showed heavy yeast growth, as expected. Fusion proteins made using Cand1, 2, 3, 5, 7, 8 and HHP2 in the NubG-candidate orientation interacted with the GPA1-Cub-PLV fusion protein as indicated by yeast growth (Table 2). The NubG TOM1 fusion protein did not interact with GPA1-Cub-PLV in our highly stringent conditions with 1 mM methionine (Figure 3e), nor did it show any interaction when the methionine concentration was reduced five-fold to 200 μM (data not shown). None of the test assays involving candidate-NubG constructs showed any interaction with GPA1, while all of the control assays showed heavy yeast growth except the assay involving HHP2, which did not show any growth (Table 2). From these data we can conclude that a free carboxyl terminus is required for Cand1, 2, 3, 5, 7, and 8 to interact with GPA1.

**Table 2:** Results for the split-ubiquitin system protein-protein interaction assays between candidate GPCR Nub fusion proteins and the GPA1-Cub-PLV fusion protein

		Candidate GPCR Nub fusion orientations			
		
Locus tested	Name	Nub_wt_X	Nub_G_X	XNub_wt_	XNub_G_
At1g57680.1	Cand1	++	++	++	-
At3g05010.1	Cand2	++	++	++	-
At3g59090.1	Cand3	++	++	++	-
At3g59090.3	Cand5	++	+	++	-
At5g18520.1	Cand7	++	+	++	-
At5g27210.1	Cand8	++	++	+	-
At4g21790.1	TOM1	++	-	++	-
At4g30850.1	HHP2	++	+	-	-

All results were recorded after 3-5 of days of yeast growth on minimal media containing 1 mM methionine to identify proteins that interact specifically with GPA1 as indicated by the extent of yeast growth. (+) indicates moderate growth while (++) indicate heavy yeast growth. Empty vector control plates did not show growth (data not shown). Nub_wt_, wild type amino-terminal half of ubiquitin. NubG, reduced affinity mutant of Nub_wt_.

All of the reciprocal interaction assays using the GPA1 Nub fusion proteins and the candidate-Cub-PLV fusion protein were negative, while all of the control assays involving either the Nub_wt_-GPA1 or the GPA1-Nub_wt _fusion proteins were positive except Cand8 (Table 3). Taken together, the results from the reciprocal assays provide further evidence that a free carboxyl terminus is required for candidate GPCR interaction with GPA1. Because the interaction of GPA1-Nub_wt _and Cand8-Cub-PLV did not show any yeast growth, the negative results for interaction between GPA1-NubG and Cand8-Cub-PLV are inconclusive (Table 3).

**Table 3:** Results for the split-ubiquitin system protein-protein interaction assays between GPA1 Nub fusion proteins and the candidate GPCR-Cub-PLV fusion proteins

		GPA1 Nub fusion orientations			
		
Locus tested (X-Cub-PLV)	Name	Nub_wt_-GPA1	Nub_G_-GPA1	GPA1-Nub_wt_	GPA1-Nub_G_
At1g57680.1	Cand1	++	-	++	-
At3g05010.1	Cand2	++	-	+	-
At3g59090.1	Cand3	++	-	++	-
At3g59090.3	Cand5	++	-	++	-
At5g18520.1	Cand7	++	-	++	-
At5g27210.1	Cand8	++	-	-	-
At4g21790.1	TOM1	++	-	++	-
At4g30850.1	HHP2	++	-	+	-

Results were recorded after 3-5 days of yeast growth on minimal media containing 1 mM methionine to identify proteins that interact specifically with GPA1 as indicated by the extent of yeast growth. (+) indicates moderate growth while (++) indicate heavy yeast growth. Empty vector control plates did not show growth (data not shown). X-Cub-PLV, candidate GPCR-Cub-PLV fusions. Nub_wt_, wild type amino-terminal half of ubiquitin. NubG, reduced affinity mutant of Nub_wt_.

### Classification of our *Arabidopsis *high ranking candidate GPCRs

Although GPCRs are highly divergent and generally have low sequence similarity, extensive study has led to the ability to categorize metazoan GPCRs into receptor families and subfamilies, and even subfamily categories [58]. Importantly, GPCR classification systems are based on the pharmacological properties of GPCR function [58]; therefore, classification of candidate GPCRs may give clues regarding their functional relatedness. The comprehensive GPCR classification software GPCRsIdentifier [50] was utilized to classify our candidate GPCRs in order to compare classifications of plant candidate GPCRs with those from metazoan systems.

As the GPCRsIdentifier method is independent of primary sequence and also does not attempt to verify a query sequence as having the typical 7TM topology of GPCRs prior to classification, we applied GPCRsIdentifier to proteins that we had previously predicted to contain 7TM domains (Figure 2a). GPCRsIdentifier was able to classify the great majority of these proteins: 94.74% of the 7TM proteins identified by TMHMM2, 90.56% of the 7TM proteins identified by HMMTOP2, and 91.52% of the 7TM sequences identified by Phobius were classifiable by GPCRsIdentifier.

We next specifically applied GPCRsIdentifier to classify our high ranking candidate GPCRs in the *Arabidopsis *proteome. All 16 of these candidates were classified as being class A GPCRs, and 12 of these were identified as belonging to the Olfactory subfamily (Table 4). GCR1 was the only sequence to be classified as belonging to the Olfactory I subfamily type category, and nine of the Olfactory classified sequences were diversely classified into the Olfactory II subfamily type category numbers 1, 2, 4, 5, 8, 10, and 13. Two of the sequences classified into the Olfactory II subfamily were classified into the FOR-like category. The remaining four *Arabidopsis *high ranking candidate GPCRs were only classified to the subfamily level: three sequences were identified as belonging to the Peptide subfamily of class A, while one sequence was classified as belonging to the Viral subfamily of class A.

**Table 4:** GPCRsIdentifier classification of the high ranking candidate GPCRs in the *Arabidopsis*, *Oryza*, and *Populus *proteomes

Genus and locus	ID	Prediction	Family	Subfamily	Subfamily type
***Arabidopsis***					
**At1g48270.1**^†^	GCR1	GPCRs	Class A	Olfactory	Olfactory I fam
**At3g26090.1**^†^	RGS1	GPCRs	Class A	Peptide	
**At1g57680.1**	Cand1	GPCRs	Class A	Viral	
**At3g05010.1**	Cand2	GPCRs	Class A	Olfactory	Olfactory II fam 4
**At3g59090.1**	Cand3	GPCRs	Class A	Olfactory	Olfactory FOR-like
At3g59090.2	Cand4	GPCRs	Class A	Olfactory	Olfactory FOR-like
**At3g59090.3**	Cand5	GPCRs	Class A	Olfactory	Olfactory II fam 8
At4g21790.1*	TOM1	GPCRs	Class A	Olfactory	Olfactory II fam 10
At5g02630.1	Cand6	GPCRs	Class A	Olfactory	Olfactory II fam 4
**At5g18520.1**	Cand7	GPCRs	Class A	Peptide	
**At5g27210.1**	Cand8	GPCRs	Class A	Olfactory	Olfactory II fam 13
At2g02180.1	TOM3	GPCRs	Class A	Olfactory	Olfactory II fam 5
**At4g30850.1**	HHP2	GPCRs	Class A	Olfactory	Olfactory II fam 1
At5g26740.1	Cand9	GPCRs	Class A	Olfactory	Olfactory II fam 4
At1g14530.1	THH1	GPCRs	Class A	Peptide	
At3g05940.1	Cand10	GPCRs	Class A	Olfactory	Olfactory II fam 2
					
***Oryza***					
*Os01g54784.1*		GPCRs	Class A	Peptide	
*Os01g61970.1*		GPCRs	Class A	Rhodopsin	
*Os01g66190.1*		GPCRs	Class C		
*Os02g40550.1*		GPCRs	Class A	Rhodopsin	
Os02g45870.1		GPCRs	Class A	Olfactory	Olfactory I fam
Os03g36790.1		GPCRs	Class A	Olfactory	Olfactory II fam5
*Os03g54920.1*		GPCRs	Class A	Rhodopsin	
*Os04g36630.1*		GPCRs	Class A	Olfactory	Olfactory II fam10
*Os04g42960.1*		GPCRs	Class A	Rhodopsin	
Os05g39730.1		GPCRs	Class A	Lysosphingolipid	
*Os06g04130.1*		GPCRs	Class A	Rhodopsin	
*Os06g09930.1*		GPCRs	Class A	Olfactory	Olfactory I fam
Os07g01250.1		GPCRs	Class A	Olfactory	Olfactory II fam4
					
***Populus***					
*Pop205267*		GPCRs	Class A	Peptide	
*Pop240991*		GPCRs	Class A	Olfactory	Olfactory II fam2
*Pop241510*		GPCRs	Class A	Olfactory	Olfactory II fam4
Pop254437		GPCRs	Class A	Peptide	
*Pop256636*		GPCRs	Class A	Olfactory	Olfactory II fam10
Pop272274		GPCRs	Class A	Thyrotropin	
Pop273474		GPCRs	Class A	Peptide	
Pop279432		GPCRs	Class C		
*Pop294952*		GPCRs	Class A	Olfactory	Olfactory II fam2
*Pop554569*		GPCRs	Class A	Nucleotide	
*Pop561523*		GPCRs	Class A	Olfactory	Olfactory II fam4
*Pop569632*		GPCRs	Class A	Olfactory	Olfactory I fam
Pop647588		GPCRs	Class A	Olfactory	Olfactory I fam
*Pop742547*		GPCRs	Class A	Olfactory	Olfactory II fam6
Pop762585		GPCRs	Class A	Peptide	
Pop796139		Globular			
*Pop797267*		GPCRs	Class A	Nucleotide	
*Pop820940*		GPCRs	Class A	Olfactory	Olfactory II fam9
Pop822025		GPCRs	Class A	Olfactory	Olfactory II fam5
Pop832788		GPCRs	Class A	Olfactory	Olfactory I fam

*Arabidopsis *candidate GPCRs shown to interact with GPA1 *in vivo *in the split-ubiquitin fusion assays are identified in bold while the sole negative result is indicated with an asterisk. *Oryza *and *Populus *candidate GPCRs that are orthologous to one of the emboldened *Arabidopsis *candidates are identified in italic type (see Table 5 for orthology details). ^†^GCR1 and RGS1 interaction with GPA1 in the split-ubiquitin system was previously described by Pandey and Assmann [60] and Chen *et al*. [20], respectively.

### Application of our GPCR detection method to the *Oryza *proteome

**Figure 4:**
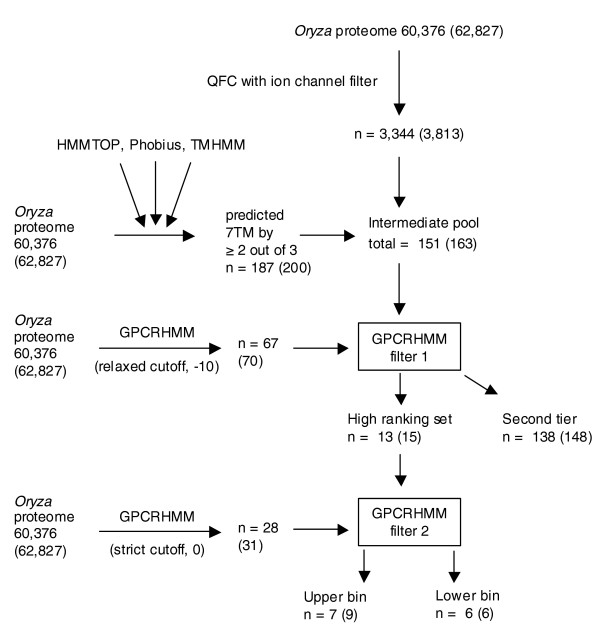
Flowchart detailing our *O. sativa *candidate GPCR identification scheme. Numbers in parentheses include redundant protein sequences. A complete list of splice variants and redundant proteins for the *Oryza *proteome is supplied in Additional data file 13.

To identify candidate GPCRs in *Oryza *the same bioinformatics pipeline was applied as was used for *Arabidopsis*. Application of the QFC algorithm to the *Oryza *non-redundant proteome identified 3,344 proteins as GPCRs. Topology predictions using TMHMM2, HMMTOP2, and Phobius identified 187 proteins that were predicted to have 7 TMs by at least two of these programs, after considering the presence of signal peptides (Figure 2b). As summarized in Figure 4, we identified an intermediate pool of 151 non-redundant *Oryza *candidate GPCRs that satisfied the criterion of direct prediction as a GPCR by the QFC algorithm and a majority 7TM topology prediction. Sixty-seven proteins in this intermediate pool were predicted to have exactly seven TM domains by consensus prediction (Additional data file 2). Application of GPCRHMM with a relaxed global threshold to the intermediate pool resulted in identification of 138 second tier candidate GPCRs (Additional data file 2) and 13 high ranking candidate GPCRs (Table 5). Seven of these sequences were further segregated into an upper bin of high ranking candidates using the additional filtering steps of requiring a GPCRHMM global score greater than 0 and a positive GPCRHMM local score (Table 5).

**Table 5:** Characterization of our high ranking *Oryza *candidate G-protein coupled receptors

Locus	QFC	GPCRHMM	TMHMM	HMMTOP	Phobius	Pcut-T	Pcut-H	Query	e-value
**Upper bin**									
**Os01g54784.1**	*	*	7 (out)	7 (out)	7 (out)			Cand3,4,5	<*e*^-85^
**Os01g61970.1**	*	*	8 (in)	9 (out)	7 (out)	7 (out)	7 (out)	Cand7	e^-150^
**Os01g66190.1**	*	*	7 (out)	7 (out)	7 (out)			Cand1	e^-65^
**Os04g36630.1**	*	*	7 (out)	7 (out)	7 (out)			Cand1	e^-60^
Os05g39730.1	*	*	6 (in)	7 (out)	7 (out)				
**Os06g04130.1**	*	*	7 (in)	9 (out)	7 (out)	6 (out)	7 (out)	Cand7	e^-123^
**Os06g09930.1**	*	*	7 (out)	7 (out)	7 (out)	7 (out)	7 (out)	GCR1	e^-120^
									
**Lower bin**									
Os03g36790.1	*	-0.81	8 (in)	8 (in)	7 (out)	7 (out)	7 (out)		
Os07g01250.1	*	-1.16	7 (out)	7 (out)	7 (out)				
**Os02g40550.1**	*	-1.17	8 (in)	8 (in)	7 (out)	7 (out)	7 (out)	Cand7	e^-111^
**Os04g42960.1**	*	-4.39	8 (in)	9 (out)	7 (out)	7 (out)	8 (in)	Cand7	e^-120^
**Os03g54920.1**	*	-8.01	7 (in)	6 (out)	7 (out)			Cand2,8	<e^-96^
Os02g45870.1	*	-9.73	6 (in)	7 (out)	7 (out)				

High ranking *Oryza *candidate GPCRs that are orthologous to our *Arabidopsis *candidate GPCRs demonstrated to interact with the *Arabidopsis *Gα subunit are shown in bold and BLAST e-values are provided to support their identification (see Table 1 for additional details).

Four of the thirteen high ranking candidates were predicted to have seven TM domains by all three TM predictors, although 7TM consensus predictions became evident for three of the sequences (Os01g61970.1, Os03g36790.1, and Os02g40550.1) only after considering the confounding effect of signal peptides on amino-terminal TM domain prediction (Table 5).

### Classification of the *Oryza *high ranking candidate GPCRs

GPCRsIdentifier classified all but one of the high ranking candidate GPCRs in the *Oryza *proteome into the class A family of GPCRs (Table 4). Interestingly, the two *Oryza *putative paralogs most closely related to Cand1 were classified differently; Os04g36630.1 was classified as belonging to the class A family while Os01g66190.1 was classified as belonging to the class C family. This may indicate that these *Oryza *candidate GPCRs have functionally diverged and have differential ligand specificities since GPCR classification systems are based on pharmacological function.

Classification of the *Oryza *class A candidate GPCRs identified a greater diversity of subfamily representation than that seen in the *Arabidopsis *analysis (Table 4). Only 5 of the 13 candidates were classified into the Olfactory subfamily and of these, two were identified as Olfactory I family sequences. The other three were classified into the Olfactory II subfamily type category numbers 4, 5, and 10. Another 5 of the 13 candidates were classified into the Rhodopsin subfamily, and the remaining 2 sequences were divided between the Peptide and Lysosphingolipid subfamilies.

### Application of our GPCR detection method to the *Populus *proteome

**Figure 5:**
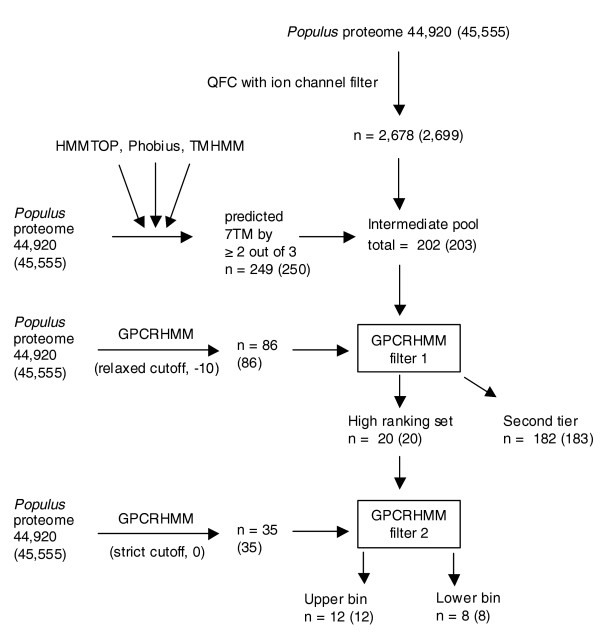
Flowchart detailing our *P. trichocarpa *candidate GPCR identification scheme. Numbers in parentheses include redundant protein sequences. A complete list of splice variants and redundant proteins for the *Populus *proteome is supplied in Additional data file 14.

Direct detection of potential candidate GPCRs by the QFC algorithm identified 2,678 sequences within the non-redundant *Populus *proteome (Figure 5) and our protein topology analysis identified a total of 249 protein sequences predicted to be heptahelical by two out of the three prediction programs (Figure 2c). The intermediate pool of *Populus *candidate GPCRs, defined as those proteins that satisfied both the QFC and majority 7TM prediction requirements, contains 202 proteins of which 96 are 7TM by prediction consensus (Additional data file 3).

Using the same GPCRHMM criteria as previously employed, we identified 20 high ranking candidate GPCRs in the non-redundant *Populus *proteome (Table 6), 12 of which compose an upper bin of candidates as they were co-predicted by the QFC and GPCRHMM using our most stringent criteria. Of these 20 high ranking candidate GPCRs, 16 are predicted to have 7 TM domains by agreement of TMHMM2, HMMTOP2, and Phobius. Nine of the consensus 7TM prediction sequences are found within the twelve sequence upper bin (Table 6).

**Table 6:** Characterization of our high ranking *Populus *candidate G-protein coupled receptors

Locus	QFC	GPCRHMM	TMHMM	HMMTOP	Phobius	Pcut-T	Pcut-H	Query	e-value
**Upper bin**									
**Pop205267**	*	*	7 (out)	7 (out)	7 (out)			Cand3,4,5	<e^-110^
**Pop241510**	*	*	7 (out)	7 (out)	7 (out)	7 (out)	7 (out)	Cand7	e^-130^
Pop254437	*	*	7 (out)	7 (out)	7 (out)	7 (out)	7 (out)		
**Pop256636**	*	*	7 (out)	7 (out)	7 (out)			Cand7	0.0
**Pop561523**	*	*	7 (out)	7 (out)	7 (out)	7 (out)	7 (out)	Cand7	0.0
**Pop569632**	*	*	7 (out)	7 (out)	7 (out)			Cand1	e^-32^
**Pop742547**	*	*	7 (out)	7 (out)	7 (out)			Cand1	e^-110^
**Pop797267**	*	*	7 (out)	8 (in)	7 (out)	7 (out)	7 (out)	Cand7	e^-129^
**Pop820940**	*	*	7 (out)	7 (out)	7 (out)			GCR1	e^-142^
**Pop272274**	*	*	7 (out)	8 (in)	7 (out)			Cand1	e^-114^
Pop554569	*	*	7 (out)	7 (out)	7 (out)	6 (in)	7 (out)	Cand7	e^-130^
Pop647588	*	*	6 (in)	7 (out)	7 (out)				
									
**Lower bin**									
**Pop294952**	*	-1.05	7 (out)	7 (out)	7 (out)			Cand2,8	<e^-115^
**Pop240991**	*	-2.37	7 (out)	7 (out)	7 (out)			Cand2,8	<e^-117^
Pop273474	*	-2.99	7 (out)	7 (out)	7 (out)	7 (out)	7 (out)		
Pop822025	*	-3.67	7 (out)	7 (out)	7 (out)				
Pop279432	*	-4.84	7 (in)	7 (out)	7 (out)				
Pop796139	*	-5.58	7 (in)	8 (in)	7 (in)				
Pop762585	*	-6.23	7 (out)	7 (out)	7 (out)				
Pop832788	*	-8.32	7 (out)	7 (out)	7 (out)				

Protein sequences orthologous to our *Arabidopsis *candidate GPCRs demonstrated to interact with the *Arabidopsis *Gα subunit are shown in bold and BLAST e-values are provided to support their identification (see Table 1 for additional details).

### Classification of the *Populus *high ranking candidate GPCRs

Out of the 20 *Populus *high ranking candidate GPCRs, 18 protein sequences were classified as class A GPCRs by GPCRsIdentifier (Table 4). Of these, eleven were classified into the Olfactory family with three identified as belonging to the Olfactory I subfamily and eight identified as belonging in one of the Olfactory II subfamily type category numbers 2, 4, 5, 6, 9, and 10. The remaining seven class A sequences were identified as belonging to the Peptide (four), Nucleotide (two), and Thyrotropin (one) subfamilies. One sequence, Pop 279432, which was not classified as a class A GPCR, was classified into class C, while the remaining non-class A sequence, Pop796139, was classified by GPCRsIdentifier as a globular protein.

### Conservation of high ranking candidate GPCRs across monocot and dicot plants and metazoa

Since individual GPCRs and GPCR families are known to be evolutionarily conserved across species [59], we sought to identify sequences in the *Oryza *and *Populus *proteomes that are homologous to our *Arabidopsis *candidate GPCRs that we empirically demonstrated to interact with GPA1 (Tables 2 and 3). Specifically, we hypothesized that our *Arabidopsis *candidate GPCRs shown to interact with GPA1 should have likely orthologs in the *Oryza *and *Populus *proteomes and that these likely orthologs should also have been predicted as candidate GPCRs using our most stringent identification scheme.

**Figure 6:**
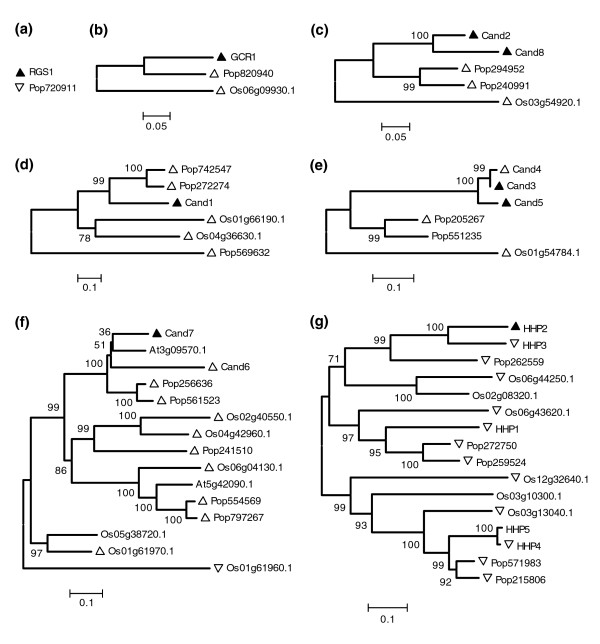
Molecular evolutionary analyses of candidate GPCRs shown to physically interact with GPA1. The *Arabidopsis*, *Oryza*, and *Populus *proteomes were subjected to BLAST analyses (e-20 cutoff) using our positive interacting candidate GPCR protein sequences (filled triangles). Multiple sequence alignments were created in ClustalX and evolutionary relationships were estimated using the neighbor joining method with 1,000 bootstrap replicates. Sequences identified by our bioinformatic pipeline as candidate GPCRs are indicated with empty triangles, with upward pointing triangles indicating those found within our high ranking candidate sets and downward pointing triangles indicating those present in the second tier. Scale bars indicate evolutionary distance as measured by residue substitutions per site. **(a) **RGS1; **(b) **GCR1; **(c) **Cand2 and Cand8; **(d) **Cand1; **(e) **Cand3, 4, and 5; **(f) **Cand6 and Cand7; and **(g) **HHP2.

To evaluate the hypothesis that our *Arabidopsis *high ranking candidate GPCRs shown to physically interact with GPA1 exhibit sequence conservation in higher plants, we performed phylogenetic analyses using potential orthologs identified by BLAST analyses of the *Arabidopsis*, *Oryza *and *Populus *proteomes (Figure 6). This molecular evolutionary analysis supported both parts of our hypothesis. First, as described in more detail below, all seven of our interacting GPCRs as well as GCR1, previously shown to interact with GPA1 [60], indeed have close homologs (E-values < e^-60^) in *Oryza *and *Populus*, while RGS1 [20] has a close homolog only in *Populus *(Tables 4 and 5, Figure 6). Second, nearly all of the orthologous sequences uncovered by phylogenetic analyses were independently predicted as GPCRs using our GPCR prediction pipeline (Figure 6), despite differences in primary sequence, physiochemical characteristics, and topological boundaries.

Using GCR1 as the input sequence, we identified single homologous proteins in both the *Oryza *and *Populus *proteomes (Os06g09930.1, Pop820940), and both these proteins were among those independently predicted by our bioinformatic pipeline as high-ranking candidates in these proteomes. Queries using the RGS1 sequence did not identify a homolog in the proteome of the monocot, *Oryza*, but did identify a sole homologous protein in the proteome of the dicot, *Populus *(Pop720911). This sole *Populus *RGS1 homolog was identified as second tier candidate GPCR by our bioinformatic analysis. Further queries of publicly available databases show that GCR1 is highly conserved across the plant kingdom, including dicots and monocots, while RGS1 sequences are highly conserved within the dicotyledonous species (data not shown).

BLAST analyses showed that Cand1 has no homologs within the *Arabidopsis *proteome, but it does have two highly similar proteins in the *Oryza *proteome and three homologs in the *Populus *proteome (Figure 6d), all of which we had previously identified as high ranking candidate GPCRs. Although highly similar sequences (BLAST < e^-95^) were identified in other plant species, the identification of non-plant possible homologs of Cand1 was limited to a single *Dictyostelium *protein [GenBank:XP_637589] with an expected value of 5e^-07 ^(data not shown).

**Figure 7:**
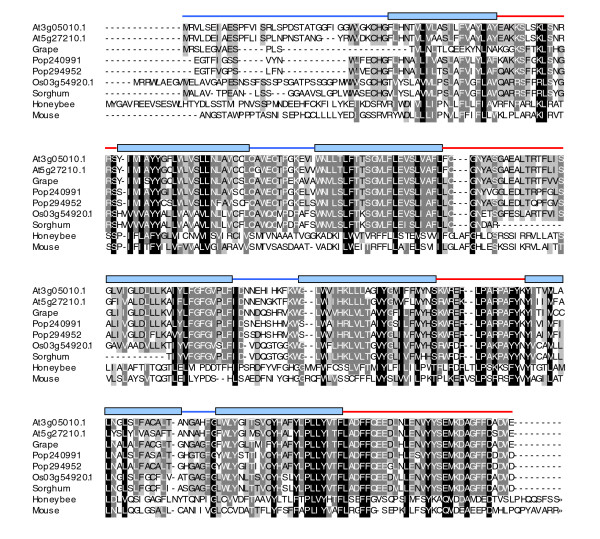
Multiple sequence alignment of the Cand2 (At3g05010.1) and Cand8 (At5g27210.1) family. The family is widely conserved beyond the *Oryza *and *Populus *proteomes; homologous sequences can be found in other dicotyledonous plants (grape [GenBank:CAN61534.1]), monocotyledonous plants (sorghum [GenBank:AAM47585.1]), insects (honeybee [GenBank:XP_625021.2]), and mammals (mouse GPR175 protein [GenBank:AAH10244.1]). The long carboxy-terminal region of the honeybee and mouse protein sequences are truncated due to the lack of any meaningful alignment beyond that shown. Schematic above the alignment blocks indicates the 7TM topology of Cand2, as predicted by TMHMM. Blue lines, extracellular regions; blue blocks, TM domains; red lines, intracellular regions.

Candidate GPCRs Cand2 and Cand8, which share 83% identity and compose a two gene family in *Arabidopsis*, identified a similarly closely related protein pair in *Populus *but only identified a single homolog in the *Oryza *proteome; we had previously identified all three of these proteins as belonging to the high ranking candidate GPCR gene sets of these proteomes. The Cand2/8 family is not only widely conserved across monocot and dicot plant lineages, but is also conserved across higher metazoa as BLAST searches identify homologs in mouse (GPR175) and honeybee (Figure 7).

Queries with all three splice variants of At3g59090 (Cand3, 4, and 5) detected a single sequence in the *Oryza *proteome (Os01g54784.1) and two sequences (Pop205267, Pop551235) in the *Populus *proteome (Figure 6e). BLAST analyses using these At3g59090 splice variants did not detect any non-plant sequences, suggesting that this family, like the TOM1/3 family with which it is weakly associated, is plant-specific.

The HHP family has five members in *Arabidopsis *and has previously been reported to be similar to human adiponectin and progestin receptors [53]. BLAST searches of the *Oryza *and *Populus *proteomes using HHP2 identify five homologs in the *Oryza *proteome and six homologs in the *Populus *proteome (Figure 6g), and all but 2 of these 11 sequences were found by our independent GPCR candidate search.

BLAST searches using Cand6 and Cand7, which compose half of a four gene family in *Arabidopsis*, identified six homologous protein sequences in the *Oryza *proteome and five in *Populus*. A broader BLAST analysis, using all of these sequences as queries, showed that the Cand6/7 'super-family' contains 29 non-redundant members within the *Arabidopsis*, *Oryza*, and *Populus *proteomes. The majority of these sequences (20/29) are independently identified by our GPCR prediction pipeline as candidate GPCRs, with 13 of the 20 sequences identified as high ranking candidate GPCRs. Molecular evolutionary analyses using all 29 members show that the superfamily strongly and equally bifurcates into two clades (Additional data file 4), with one clade containing Cand6, Cand7, and all of their close homologs identified in the initial BLAST analyses (Figure 6f). Subsequent BLAST searches using Cand7 as the query show that this large plant family of sequences is similar to the human GPR107 family of GPCRs and conserved across plants, insects, fish, and mammals (Additional data file 5 and data not shown).

To further characterize the phylogenetic relationships identified by our molecular evolutionary analyses, we queried the Pfam database [52] using all of our candidate GPCRs (Additional data file 6). Domain search analyses using the Pfam database confirm the previous descriptions of GCR1 having a Dicty_CAR domain, and we find that this attribute is also found in the *Oryza *and *Populus *homologs of GCR1 (Figure 6; Additional data file 6). Our analyses show that the plant Cand6/7 superfamily members all contain the Lung_7TMR domain (Figure 6; Additional data file 6), which is common to the mammalian GPR107/108 family. Plant sequences in the TOM1/3 family have a domain of unknown function, DUF1084, while Cand9 and Cand10 both have a DUF300 domain. The haemolysin-III domain of the HHP2 family of *Arabidopsis *sequences was previously noted [53] and we show that this domain is conserved across the greater HHP family in *Oryza *and *Populus *(Figure 6; Additional data file 6). Interestingly, several of our candidate GPCRs and candidate GPCR families (Cand1-5 and Cand8) do not have any of the domains included in the PfamA database. This provides additional support that these are novel, uncharacterized proteins, but does not provide negative support for their identification as a candidate GPCR: analysis of all of the human sequences available in the GPCRDB using the Pfam database shows that 21.4% do not have any associated PfamA domains, and 6.2% of the sequences have domains that are other than those annotated as GPCR specific (data not shown). Similar to the number observed for the human GPCRDB sequences, 20.4% of our *Arabidopsis *candidate GPCRs did not have matches in the PfamA database. Although PfamB family domains are not annotated and are of lower quality, all of our candidate GPCRs without PfamA domains were assessed for the possibility of functionally conserved domains in order to computationally characterize these proteins to the fullest extent. After PfamB analyses, we find that nearly all of these candidate GPCRs have some type of uncharacterized domain (Additional data file 7). Interestingly, in some cases the associated domain is based exclusively on data from members of the candidate GPCR family or superfamily. For instance, members of the Cand2/8 family have Pfam-B_26759 and Pfam-B_14631 domains, but these domains are based on the ProDom alignment of Cand2/8, a sorghum homologue, and the human GPR175 sequence (see also Figure 7). This domain analysis suggests that these two unannotated PfamB domains may be uncharacterized GPCR domains, but this remains to be proven.

Taken together, our results show that the high ranking *Arabidopsis *candidate GPCRs that we have empirically shown to interact with GPA1 are widely conserved in plant species, and that homologous sequences in other plant proteomes are indeed independently predicted as high ranking GPCRs by our approach, further supporting the validity of this method.

## Discussion

### Bioinformatic identification of *Arabidopsis *candidate GPCRs

The experimental elucidation of candidate plant GPCRs has so far been limited to the discovery of *Arabidopsis *GCR1 [25] and its homolog in pea [61], *Arabidopsis *RGS1 [28], and, recently, *Arabidopsis *GCR2 [26]. Within the *Arabidopsis *genome no other genes have any appreciable similarity to GCR1 or RGS1 by BLAST analysis. GCR2 and its two homologs within the *Arabidopsis *genome are homologous to the lanthionine synthetase C family [27], and furthermore, all of the key LanC-like family GXXG motifs as well as the catalytic residues are conserved between GCR2 (At1g52920.1) and lantibiotic cyclase, for which a crystal structure is known [PDB:2g02, PDB:2g0d] [62]. Although GCR2 was reported by Liu and co-workers [26] as a GPCR, none of the 16 TM prediction programs used to create the ARAMEMNON membrane protein database [63] predict this protein to have seven TM domains, including DAS and TM-PRED, which were included in the Liu *et al*. report [26]. Our whole proteome analysis using our multiple topology prediction approach did not predict a single TM domain within this protein. Illingworth *et al*. [62] mathematically describe how GCR2 can be misconstrued to have transmembrane domains and show that GCR2, similar to other lanthionine synthetases, does have short hydrophobic stretches but these short regions encompass the conserved GXXG motifs and map to a single face of the 2g02 crystal structure. Interestingly LANCL1, another lanthionine synthetase, was initially identified as a GPCR [64] prior to biochemical experimentation, which confirmed its subcellular localization as a peripheral membrane protein. Additional discrepancies have also arisen regarding the description of GCR2 as a GPCR that functions as a receptor for the plant hormone abscisic acid. Gao *et al*. [27] report that GCR2 is not genetically or physiologically coupled to GPA1 and is not required for abscisic acid perception during seed germination and seedling development

GCR1 has no homologs within the *Arabidopsis *proteome. BLAST searches of other plant proteomes, including *Oryza *and *Populus*, do readily identify sequences highly similar to GCR1, but subsequent BLAST searches using these identified putative orthologs of GCR1 suggest that these genes also have no homologs within their respective proteomes. The lack of obvious homologs of GCR1 in each proteome precludes the ability to discover new potential GPCRs through the use of simple homology-based searches. Attempts to identify plant candidate GPCRs through the use of publicly available GPCR specific databases were also not productive; the GPCRDB database [58] and the SEVENS database [65] contain only GCR1 and sequences from the mildew locus o (MLO) family, although SEVENS also includes GCR2 and its two homologs. Searches of the GPCR/G-protein/effector database gpDB [66] did not identify any plant sequences in the GPCR category.

To circumvent these problems, we have developed a combinatorial approach to identify novel GPCRs based on the direct prediction of GPCRs by the QFC algorithm and GPCRHMM; signal peptide detection by Phobius; TM domain prediction by TMHMM2, HMMTOP2, and Phobius; and subsequent GPCR classification by GPCRsIdentifier. Our bioinformatic analyses of the *Arabidopsis *proteome identified a primary tier of 16 high ranking candidate GPCRs using the criteria that sequences were required to be co-predicted as a GPCR by the QFC algorithm and GPCRHMM and have a predicted 7TM topology by at least two of the transmembrane prediction programs.

Notably, both GCR1 and RGS1, two proteins experimentally confirmed to functionally couple to the sole Gα subunit in *Arabidopsis *[20,60], are found within our primary tier of candidate GPCRs (Table 1). RGS1, which has both a 7TM domain and a long carboxy-terminal RGS domain, was directly predicted as a GPCR by GPCRHMM only when analyses were performed using the 7TM domain of the protein. This is because inclusion of the carboxy-terminal RGS domain introduced sequence bias from the intrinsic amino acid composition and dipeptide frequency of this domain, resulting in a lower Global score of -15.27.

Also found in this primary tier of GPCR candidates is HHP2, one of five members of the *Arabidopsis *HHP family with sequence similarities to human adiponectin receptors and membrane progestin receptors [53], and two members of the TOM1/3 family implicated in tobamovirus multiplication [54]. Of the 16 proteins in our primary tier, seven have not been previously studied and are only annotated as expressed proteins. The inclusion of these biologically uncharacterized proteins in our candidate GPCR list provides both a clue as to their function and a framework to guide the design of future experimental work.

Since GPCRHMM appears to be highly specific, or at least highly conservative, in identifying novel plant GPCRs, we reasoned that our strict criteria for identifying the highest ranking sequences most likely excluded the identification of potentially useful candidates. Removal of the high ranking candidates identified by GPCRHMM from the intermediate pool led to the identification of 111 second tier candidate GPCRs, including HHP1, HHP3, and three members of the MLO family: MLO7, MLO10, and MLO13. The plant-specific MLO family is named after a barley MLO protein that was experimentally shown to have a 7TM GPCR-like topology and play a key role in mediating fungal infection [67]. Aside from the 7TM topology, there is no evidence to suggest that any MLO family members couple to Gα. Additional MLO family members (MLOs 2, 3, 4, 6, 8, 11 and 14) are identified by our QFC analysis but are subsequently removed by our ion channel filter step (Additional data file 8).

Attempts to reconstruct an overall evolutionary relationship using our 16 high ranking candidate GPCRs proved fruitless, and the inclusion of the remaining set of 111 candidate GPCRs did not provide any greater resolution beyond the obvious small gene family clusters identified by BLAST analyses of the *Arabidopsis *proteome alone (Figure 6). These phylogenetic results were expected based on the lack of sequence homology between our candidate GPCR clusters, which mirrors the well-established lack of a comprehensive phylogenetic relationship linking all metazoan GPCRs of an organism.

### Prediction of candidate GPCR coupling specificity

In the human system, the heterotrimeric G-protein contains one of 23 different Gα subunits and some GPCRs are described as promiscuous because they can couple to more than one type of Gα subunit. Although *Arabidopsis *contains only one canonical Gα subunit, GPA1, which is most similar to a G_z _variant of the G_i/o _subunit family [3], we used Pred-Couple 2 to predict the coupling specificity of our candidate GPCRs. Since GPA1 belongs to the G_i/o _subunit family, it follows that *Arabidopsis *candidate GPCRs associated with GPA1 should be predicted to couple with members of the G_i/o _family. Our analyses show that 92.2% of our *Arabidopsis *candidate GPCRs for which Pred-Couple 2 provides a coupling prediction are indeed predicted to couple to the G_i/o _family (Additional data file 9). Note that the absence of a coupling prediction does not indicate that a sequence is not a GPCR, because Pred-Couple 2 initially filters sequences using parameters based on established GPCRs and is not, therefore, designed to detect novel or divergent GPCRs [51].

### *In vivo *testing of protein coupling

With the information provided by our bioinformatic analyses, we turned towards providing empirical evidence that some of our top candidates truly have the potential to function as a GPCR. The split-ubiquitin system, a membrane-based variant of the yeast two-hybrid assay, has been used to demonstrate coupling of the candidate GPCRs, GCR1 and RGS1, to the sole *Arabidopsis *Gα subunit, GPA1 [20,60]. GPA1 has been shown to act specifically in this binding assay as it does not bind the inward potassium channel KAT1 or a truncated version of GCR1 [60]. Our *in vivo *protein-protein binding experiments demonstrated that the great majority (7/8) of the candidate GPCRs that we tested do interact with GPA1. We show that candidates Cand1, 2, 3, 5, 7, 8, and HHP2 all couple to GPA1, provided that the carboxyl terminus of the candidate is not blocked by a fused protein tag. This requirement for a free carboxyl terminus was observed previously for GCR1 [60]. TOM1 did not interact with GPA1 in any of our assays regardless of protein fusion orientation. Given the apparent specificity of GPA1 in the split-ubiquitin system, our positive protein-protein interactions now await confirmation of interaction *in planta*. The ability to not only bind Gα but to stimulate the exchange of guanosine diphosphate for guanosine triphosphate is a key characteristic of classically functioning GPCRs that could also be assessed in future studies.

Within the *Arabidopsis *proteome, the candidates we tested for *in vivo *coupling to GPA1 ranged from a single gene to members of small families. Cand1 has no homologs within the proteome, candidates Cand2 and Cand8 comprise a small two gene family, Cand3 and Cand5 are two splice variant products of the same locus, and Cand7 is a member of a small four gene family in *Arabidopsis*. Interestingly, Cand6 (At5g02630.1), which is the second closest homolog to Cand7, is also identified in our top tier of candidate GPCRs, suggesting that the other two members of this family may also couple to GPA1. HHP2 is part of the five gene HHP family [53], and we predict all but HHP5 to be GPCRs, suggesting these sequences also may compose a GPCR family.

Given the positive correlation between our high stringency computational analysis identifying candidate GPCRs and our subsequent *in vivo *assay showing physical interaction with GPA1 under high stringency (1 mM methionine) conditions [68], it is likely that these proteins, and their close homologs, actually function as GPCRs.

### Bioinformatic method application to the *Oryza *and *Populus *proteomes

As our method for identifying novel plant candidate GPCRs successfully identified a set of *Arabidopsis *high ranking proteins, most of which were demonstrated to physically couple with GPA1, we next applied our method to the proteomes deduced from the fully sequenced *Oryza *and *Populus *genomes. Our analyses identified 13 and 20 high ranking candidate GPCRs, and an additional 138 and 182 second tier candidate GPCRs in the *Oryza *and *Populus *proteomes, respectively. Similarly as described for our *Arabidopsis *candidate GPCRs, those *Oryza *and *Populus *candidates for which coupling predictions were obtained using Pred-Couple 2 were primarily predicted to couple to the G_i/o _type of Gα subunit (Additional data file 9). And for *Oryza*, in which the Gα subunit has been characterized, our results are consistent as the *Oryza *Gα subunit shows sequence similarities to subunits of the human G_i _family (data not shown).

### Evolutionary dynamics of candidate GPCRs in plants

One hallmark of metazoan GPCRs is the conservation of individual GPCRs across divergent organisms. BLAST analyses using the *Arabidopsis *candidate GPCRs that we experimentally demonstrated to couple with GPA1 in the protein binding assays identified a number of homologous sequences in the *Oryza *and *Populus *proteomes, the great majority of which (47/53) were also independently predicted as GPCRs by our bioinformatic method (Figure 6). With the exception of HHP2, all of the *Arabidopsis *candidate GPCRs that we demonstrated to couple with GPA1 have homologs within both the *Oryza *and *Populus *top tier candidate lists.

The small *Arabidopsis *gene family of Cand2 and Cand8, which shows approximately 22% identity, and approximately 43% similarity to the mammalian GPR175 GPCR family, has a corresponding two gene family in *Populus*, but only a single homolog is identifiable in the *Oryza *proteome, suggesting a potential evolutionary loss. Additional homologs can be identified within other plant (grape and sorghum), insect (honeybee), and mammal (human, mouse, rat) predicted proteomes. Multiple sequence alignments show that the intracellular carboxyl terminus, which has been described as the 'magic tail' due to its ability to couple with multiple GPCR-interacting proteins including Gα [57], has near complete identity (Figure 7) within the group of plant sequences. This high sequence conservation across diverse plant genera points towards conserved binding partners and potential signaling mechanisms.

In contrast to the small number of sequences that compose the other high ranking candidate GPCR families, the *Arabidopsis *candidate GPCR Cand7 belongs to a small gene family, which, in turn, belongs to a large 29 member protein 'superfamily' found within the *Arabidopsis*, *Oryza *and *Populus *proteomes. Phylogenetic analyses show that the Cand6/7 superfamily deeply bifurcates to form two distinct clades. One clade contains the three closest *Arabidopsis *homologs of Cand7 (including Cand6), and the other clade contains three only distantly related *Arabidopsis *sequence (Additional data file 4). The two clades are linked by a single *Arabidopsis *sequence found at the midpoint of the reconstructed tree. The divergence of the *Arabidopsis *sequences exemplifies the difficulty of finding potential GPCRs by homology alone; without the results from our independent GPCR prediction pipeline these distant *Arabidopsis *homologs, which are phylogenetically surrounded by candidate GPCRs from the *Oryza *and *Populus *proteomes, would not have been discovered as belonging to the Cand6/7 superfamily. All of the proteins within the Cand6/7 superfamily contain a Lung 7TM receptor domain (Additional data file 6) and are related to the GPR107/108 orphan GPCR superfamily that contains the same domain. Interestingly, one residue, Cand7 Trp^193^, located near the interior membrane junction of TM1, is invariant in all 29 non-redundant sequences of the 3 plant proteomes, and this conservation extends across kingdoms to almost all members of the greater GPR107/108 family identified, including insects, fish, and mammals (Additional data file 5), suggesting its functional importance.

Overall, while most of our first tier candidates have homologs across the three proteomes as well as other taxa, the distribution pattern of putative orthologs and putative paralogs is heterogenous. As can be seen in Figure 6a-g, the gene trees of homologs show diverse patterns with none of the seven trees in Figure 6 showing a consistent set of putative orthologs/paralogs across the three proteomes. Thus, it seems likely that while each species retained many of the ancestral GPCRs, each seems to have specialized through both gene duplications and gene losses.

Although GPCR prediction algorithms all use sequence-derived information as a starting point, sequence homology is not the key component in our method of candidate GPCR identification. For example, our analysis identifies Cand7 and its second closest homolog, Cand6, as candidate GPCRs, but not At3g09570.1 and At5g42090.1, Cand7's first and third closest homologs. Both of these 'un-chosen' sequences were excluded from our intermediate pool by the QFC, but they were directly predicted to be GPCRs by our GPCRHMM analyses and did pass our '2/3' topology prediction requirement. The four other *Arabidopsis *sequences identified in the Cand6/7 superfamily are surrounded in the phylogenetic tree by candidate GPCRs from both *Oryza *and *Populus *and would have been candidate GPCRs had we not applied the QFC ion channel filter (Additional data file 4; Table 4). It was deemed more valuable to include rather than discard the ion channel filter (see Materials and methods), because this filter removes a large number (70) of protein sequences annotated as having channel or transport activity, 43 of which have already been identified and named, while only excluding 19 potential second tier proteins (Additional data file 8).

Although our bioinformatic approach identified a number of candidate GPCRs within each of the three proteomes analyzed, the sequences identified do not compose a homogenous group. Using the primary sequence independent four-level GPCR classification system in GPCRsIdentifier [50], we show the majority of our primary tier candidate GPCRs appear to be most similar to the class A family of GPCRs, the most abundant type of metazoan GPCRs [16], but belong to a wide range of subfamilies and subfamily types. Furthermore, the subfamily classification distribution varied between proteomes. The majority of the *Arabidopsis *primary tier sequences were classified into the Olfactory subfamily while the *Oryza *and *Populus *primary tier of candidate GPCRs showed greater diversity in amino acid composition and dipeptide frequency.

The direct meaning of these classifications is unclear relative to their descriptive names since, for example, plants do not possess an olfactory system. The GPCR classifications provided by GPCRsIdentifier may simply provide a ready-made system to catalog the breadth and diversity of plant GPCRs, and eventually, new plant-centric descriptive names should be devised for these families and subfamilies. Alternatively, these results may suggest an evolutionary relationship and indicate that mammalian GPCRs and plant GPCRs are derived from a common class of ancient GPCRs. Along these lines, it is known that some of the mammalian GPCRs bind plant secondary metabolites; for example, the ligands of opiate and cannabinoid receptors were first identified as plant-derived compounds, and only later it was discovered that mammals themselves manufacture analogous compounds: the endorphins [69] and the endocannabinoids [70]. In fact, the previously thought plant-specific compound morphine is now know to be biosynthesized *de novo *by humans [71], and morphine as well as its biosynthetic precursors have been shown to activate Gα subunits through GPCR signaling [72]. These data further suggest an evolutionary link between mammalian GPCRs and plant GPCRs.

Just as relevant evolutionary and physiological links exist between our candidate plant GPCRs and human GPCR function, plausible links also exist between plant and insect receptors. Herbivory induces plant production of volatile compounds and one such compound, methyl salicylate (MeSA), is a mobile signal that induces plant defenses in spatially distant organs of the plant under attack as well as in neighboring plants [73,74]. MeSA also activates unique neuron specific receptors of the cabbage moth *Mamestra brassicae *[75] and females of that species avoid ovipositing on plants and artificial plants equipped with MeSA emitting dispensers [75], apparently in an attempt to avoid plants already occupied by herbivores. In contrast, volatiles from herbivore-damaged plants attract wasps that parasitize insect herbivores [76], and MeSA has been shown to attract, as well as elicit electrophysiologically significant responses in, lady beetles [77], which are predators of aphids and plant mites. Homology modeling and ligand docking simulations [78,79] using our plant candidate GPCRs, predicted insect receptors, and tentative ligands such as MeSA may be helpful in identifying prospective receptors that respond to the same ligand, for example, MeSA, in both plants and insects.

### Comparison to previous plant GPCR prediction attempts

The study of heterotrimeric G-protein signaling in metazoan systems is mature and, as a result, most of the bioinformatic analyses of the GPCR family of signaling proteins are based on metazoan proteins and are designed to predict metazoan GPCRs. In comparison to both wet-bench and computational researchers studying mammalian systems, researchers in plant laboratories have relatively little information with which to identify novel candidate GPCRs. To our knowledge there have been only three published attempts at predicting GPCRs in plants, in papers by Fredriksson and Schioth [16], Inoue *et al*. [80], and Moriyama *et al*. [49].

Fredriksson and Schioth applied a hidden Markov model (HMM) approach. Although published in the year 2005, the GPCR prediction attempt by Fredriksson and Schioth [16] was performed on a pre-genome sequencing NCBI Genscan data set containing only 6,600 *Arabidopsis *predicted proteins [16] compared to the current 29,988. Their analyses identified only GCR1 and five of the MLO family proteins as GPCRs in the *Arabidopsis *data set. The identification of GCR1 is not surprising as the GCR1 protein sequence was already shown to have sufficient similarity to sequences from several classes of GPCRs to be identified as a GPCR by BLAST and PSI-BLAST analyses [81]. The identification of five *Arabidopsis *MLO sequences as GPCRs is also not surprising as Fredriksson and Schioth [16] utilized HMMs trained on the highly similar MLO family [82]. Fredriksson and Schioth [16] did extend their prediction attempts to another plant system consisting of 2,400 predicted proteins from the incomplete *Oryza *proteome, and this analysis identified only a single protein, an MLO, as a GPCR. It should be noted that the plant-specific MLOs have been described as GPCRs based solely on their 7TM topology and there are no genetic, physiological, or biochemical experimental data to support their identification as GPCRs. In fact, the one experimental test of Gα coupling, with barley MLO1, yielded negative results [67].

During the course of our study, we examined another HMM-based approach, PRED-GPCR [83], but this approach was ultimately excluded from our final analyses. PRED-GPCR utilizes a homology-oriented probabilistic approach based on identifying query sequence similarities to descriptive GPCR family-specific 'signature' motifs. Profile HMM GPCR family signatures were derived from low entropy regions of multiple sequence alignments based on GPCRs identified in the Swiss-Prot and TrEMBL databases and sorted into families. Notably, this approach does not explicitly use or assume any topological information.

Our PRED-GPCR analysis of the version 6 *Arabidopsis *proteome using the default settings identified only seven sequences (Additional data file 10). Because PRED-GPCR is based on multiple sequence alignment profile HMMs, relaxing the default settings may allow for identification of candidate GPCRs that are evolutionarily divergent from the previously identified GPCRs within the PRED-GPCR training set. Using relaxed user defined settings increased our candidate list to 19 non-redundant sequences (Additional data file 10), and the results for the PRED-GPCR default and user defined setting analyses have only one sequence in common (At4g19050.1). None of these PRED-GPCR predicted sequences were identified by our GPCR prediction pipeline. This was due to their identification as a non-GPCR by the QFC algorithm, either with or without the ion channel filter, and their predicted non-7TM topology, with the exception of At2g36630.1, which was predicted as a GPCR by the QFC but has 9 or 11 predicted TM domains, and At1g52780.1, which was not predicted by the QFC and was predicted only by Phobius to have 7 TM domains. The remainder of the PRED-GPCR predicted sequences have 0-3 or 23-24 TM domains. None of the 19 PRED-GPCR predicted sequences were identified as a GPCR by GPCRHMM.

The apparent inability of PRED-GPCR to identify *Arabidopsis *candidate GPCRs may reflect the fact that PRED-GPCR was developed and trained using a data set composed of only class B, C, D, and F GPCR sequences with a high relative proportion of sequences coming from class F, the frizzled/smoothened group. By contrast, our classification analyses using GPCRsIdentifier [50] identifies nearly all of our high ranking candidates in all three plant proteomes as class A GPCRs (Table 4), and our whole proteome analyses suggests class A type candidate GPCRs comprise the majority in plants (data not shown). This comparison provides a rationale for why these proteins were not identified by the PRED-GPCR methodology, and indicates that HMM-based approaches will prove more useful in plants when retrained using plant-specific HMMs derived from candidate plant GPCRs verified to couple with Gα, such as those identified in the present report.

In 2004, Inoue *et al*. [80] described the binary topology pattern (BTP) approach and applied it to the analyses of several proteomes. The BTP method [80] is entirely different from the QFC and HMM-based approaches in that it does not directly use any primary sequence information. The BTP method is based on the observation that although the sequences of extra-transmembrane regions (the loops and tails) of GPCRs are highly variable, there is a tendency for the lengths of these regions to be similar within GPCR families. By an iterative process, Inoue *et al*. [80] coded the extra-transmembraneous regions of known GPCRs as having a short (0) or long (1) length and found that a binary code representation of protein topology (for example, 10011001) could be used for GPCR identification and classification.

The BTP data set published by Inoue *et al*. [80] was derived from the August 13, 2001 release of the *Arabidopsis *proteome (25,542 sequences) and most predictions are invalidated by the subsequent high quality refinements of the *Arabidopsis *proteome. Examination of the *Arabidopsis *candidate GPCR data set predicted by Inoue *et al*. [80] using the BTP method showed that only 57 of the 100 predicted candidates had sequences that remain identical to a protein sequence in the current version (v7.0) of the *Arabidopsis *proteome. An additional 8 sequences have 100% identity over the aligned region, but have protein lengths that differ from the current sequence. We discount these because the BTP method is based on coding residue segment lengths. One BTP predicted GPCR sequence was identified by Inoue *et al*. as At1g42560, but actually shows the highest identity (86%) to At2g33670.1. A comparison of the still-valid 49 BTP-predicted sequences with our candidate GPCR data set shows that there are 11 sequences in common. Most notably, GCR1 and Cand7, both found within our high ranking candidate GPCR set, are identified by the BTP method. The BTP identification of GCR1, which has previously been shown to couple to GPA1 [60], and Cand7, which we show in the present study to couple to GPA1, indicates they have true GPCR topological characteristics beyond their heptahelical nature and provides further computational support for their identification as likely GPCRs. It would be interesting to see how the method of Inoue *et al*. [80] would perform on the current proteome; however, the BTP code was not made available.

The study performed by Moriyama *et al*. [49] is the most recent attempt at predicting GPCRs in *Arabidopsis*. Moriyama *et al*. [49] utilized multiple alignment free computational methods, along with TM prediction by HMMTOP2, to identify 394 sequences with predicted 5-10 TM domains. Although Moriyama *et al*. [49] further constricted this set to 54 sequences by a 7TM prediction by HMMTOP2, reliance on a single TM predictor can lead to both false positives and false negatives. Combinatorial approaches have been shown to greatly increase discrimination of 7TM sequences [84] because topology prediction programs' strengths and weaknesses vary, even in the top rated topology prediction programs [39]. Importantly, other GPCR prediction studies, including the analysis by Moriyama *et al*. [49], often failed to utilize signal peptide prediction to account for the confounding effect of signal peptides on TM domain prediction [37]. We found 6,739 non-redundant membrane proteins in the *Arabidopsis *proteome using Phobius, of which 1,209 also had predicted signal peptides. Had we also not accounted for signal peptide misprediction, we would have mistakenly discarded 2/11 proteins from our upper bin of high ranking GPCRs, including Cand7, which does physically couple with GPA1.

Although we report the predicted amino terminus location of our candidate GPCRs, and nearly all of our high ranking candidate GPCRs do indeed have a predicted extracellular amino terminus, we differ from Moriyama *et al*. [49] in that we did not specifically integrate that information into our GPCR prediction pipeline. However, had we integrated this criterion, our high ranking candidate lists would not have changed significantly (data not shown).

Our use of the alignment-free HMM GPCRHMM is another methodological difference from Moriyama *et al*. [49]. Another machine learning approach, an alignment based support vector machine method, SAM, was utilized by Moriyama *et al*. [49], but the results were not used to select their broad list of candidates as that method was found to have insufficient predictive power: SAM identified only GCR1 and 14 sequences from the 15 member *Arabidopsis *MLO family as candidate GPCRs. In contrast, we utilized the apparent high specificity of the GPCRHMM software in two serial filtering steps to identify candidate GPCRs with increasing stringency. These steps were exceedingly important as our focus went beyond computational analyses towards selecting candidate GPCRs for our functional analyses. Our GPCR prediction pipeline, which ended with our high stringency GPCRHMM filter, enabled the identification of 11 target sequences out of 29,988 non-redundant sequences in the *Arabidopsis *proteome.

Of the 394 sequences listed in Moriyama *et al*.'s [49] larger data set of possible 7TM putative receptors, we found 18 sequences that are actually redundant with other sequences and four sequences that are no longer found within the current version (v7) of the proteome. Comparing our high ranking candidate data set to Moriyama *et al*.'s high priority list shows that we predict only 14.8% (8/54) of their list to be GPCRs, and their list is missing half of our high ranking candidate GPCRs (Additional data file 11). Comparing our complete set of 127 candidate GPCRs with Moriyama *et al*.'s remaining present and non-redundant 372 sequences shows a similar story as there are only 63 sequences in common; we predict only 16.9% of Moriyama *et al*.'s list to actually be GPCRs (Additional data file 11). Perhaps this is due to a difference in research focus as Moriyama *et al*. attempt to cast the broadest net possible while identifying candidate 7TMpRs while we attempt to find the most highly likely candidate GPCRs.

Although there is overlap between our high ranking candidate GPCRs and Moriyama *et al*.'s high priority list, there are some interesting differences between the two, especially in light of our *in vivo *coupling results. The list of highest priority candidates identified by Moriyama *et al*. [49] includes Cand8, but not its closest homolog, Cand2; in fact, Cand2 is not identified by Moriyama *et al*. [49] as a candidate GPCR even using their broadest definition. Likewise, the method of Moriyama *et al*. [49] lists Cand3 as a high priority candidate GPCR but fails to identify the highly similar splice variant Cand5. We have shown here that all four proteins do in fact physically couple with GPA1. We also show by direct biological experimentation that Cand7 and HHP2 also interact with GPA1; these proteins are found only in Moriyama *et al*.'s broader list of nearly 400 candidates. This suggests the power and focus of our high stringency combinatorial analyses.

Biologically, GPCRs are interesting because of their omnipresence in metazoa and their physiological importance, while computationally, the GPCR family is interesting due the extreme range of sequence divergence, which provides an interesting case for testing the limits of bioinformatic prediction. GPCR signaling via the heterotrimeric G-protein in *Arabidopsis *is especially interesting because the G-protein complex contains only single canonical Gα and Gβ subunits, which leads to the obvious question as to whether the complement of *Arabidopsis *candidate GPCRs is similarly limited. Our data now provide an answer to this question as we show, using the same protein-protein coupling assay used for GCR1 and RGS1, that at least seven additional candidate GPCRs are present in *Arabidopsis*. Although we provide evidence showing the physical coupling of these heptahelical proteins to GPA1, we follow the convention of the GPCR community and still call these proteins candidate GPCRs to reflect the fact that a signaling ligand has not yet been identified and they, therefore, cannot unequivocally be called GPCRs. To date, this is also the situation for GCR1, and RGS1, as well as for the GCR1 homolog in pea [61].

While our study appears highly specific, it is complemented by the efforts of Moriyama *et al*. [49] and Inoue *et al*. [80], who used different prediction methods. The combinatorial approach has strength in that it considers diverse information sources before arriving at a conclusion, and thus further combination of these three independent studies should provide an even greater level of confidence that the intersecting sets of predicted GPCRs are truly G-protein coupled receptors.

## Conclusion

We have used a combinatorial approach to identify novel GPCRs based on the direct prediction of GPCRs by the QFC algorithm and GPCRHMM; signal peptide detection by Phobius; transmembrane domain prediction by TMHMM2, HMMTOP2, and Phobius; and subsequent GPCR classification by GPCRsIdentifier and coupling specificity prediction by Pred-Couple 2. After identification of candidate GPCRs, we bridged the gap between computational biology and wet-bench biology by experimental demonstration that the majority of our upper bin high ranking GPCRs, as well as the one lower bin high ranking GPCR that we tested, can physically interact with the Gα subunit of the *Arabidopsis *heterotrimer. Notably, this extension to wet bench analysis was not performed in the previous plant GPCR prediction attempts, and is rarely, if at all, performed in bioinformatic studies predicting novel GPCRs in metazoans. With experimental evidence in hand to validate our method, we classified our high ranking candidate GPCRs to examine their possible functional diversity using a non-linear sequence dependent method and examined our candidates for annotated functional protein domains. Additionally, our within-proteome and cross-proteome molecular evolutionary analyses show that our high ranking candidate GPCRs are evolutionarily conserved and that our method can be used not only to identify individual candidate GPCRs but also to identify evolutionarily conserved candidate GPCR families. Some high ranking candidate GPCRs and GPCR families are uniquely conserved within plants, while others show evolutionary conservation that extends to metazoans. These evolutionary relationships reinforce the probable functional importance of the candidate GPCRs that we have identified, and the present study is the first step towards determining their physiological roles in G-protein signaling.

## Materials and methods

### Sequence and annotation acquisition

All *A. thaliana *sequences were obtained from The *Arabidopsis *Information Resource (TAIR) ftp Gene download site [85]. All our bioinformatic analyses performed on *Arabidopsis *sequences were performed using protein sequence from the updated TAIR ATH1 version 7.0 annotation of the genome, except for those performed using PRED-GPCR, which was performed on version 6.0. Although the TAIR ATH1 annotation of the *Arabidopsis *genome advanced from version 7 to version 8 during manuscript review, none of the sequences of our *Arabidopsis *candidate GPCRs changed and our predictions are still valid. All *O. sativa *sequences were obtained from The Institute for Genomic Resource (TIGR) and downloaded from the pseudomolecules ftp site [86]. All *Oryza *bioinformatic analyses were performed on the TIGR release 5 of the Osa1 Rice Pseudomolecules and Genome Annotation database. All *P. trichocarpa *sequences were obtained from the DOE Joint Genome Initiative (JGI) and downloaded from the ftp data download site [87]. All *Populus *bioinformatic analyses were performed on the JGI version 1.1 release of the proteome. All three proteomes were the most currently available versions at the time of analysis.

### Locus abbreviations for the *Oryza *and *Populus *proteomes

For brevity, the official locus identifiers used in the *Oryza *and *Populus *proteomes have been abbreviated. For the *Oryza *data set, the locus identifier has been shortened by removing the characters 'LOC_' prior to each loci (for example, Os01g01010.1 corresponds to LOC_Os01g01010.1). For the *Populus *data set, the locus identifier has been shortened to a three letter abbreviation to indicate the *Populus *proteome followed by the unique numerical identifier for each sequence (for example, Pop171407 corresponds to jgi|Poptr1_1|171407).

### Computational analyses

To identify candidate GPCRs, bioinformatic analyses were performed with software designed to directly predict putative GPCRs, to predict protein topology, to predict the presence of signal peptides, and to classify putative GPCRs into family, subfamily, and type.

Computational analyses to directly predict candidate GPCRs were initiated by analyzing the complete proteome of *Arabidopsis *with the QFC algorithm [44], GPCRHMM [48], and PRED-GPCR [83]. The QFC algorithm from Kim *et al*. [44,88] was run using the default feature set and discriminant cutoff values; the results were further filtered by a discriminant function for ion channels based on amino acid usage frequency difference between GPCRs and channel proteins (J Kim, unpublished data). Analyses using GPCRHMM were performed with the local scoring option turned on. The *Arabidopsis *proteome version 6.0 was independently analyzed twice with PRED-GPCR. The first analysis was performed with the default parameters and the second analysis was performed with a less stringent user defined filtering option: combined family motif off, Global E-Value motif cutoff set to 1.1, and CAST low complexity filtering off. The *Oryza *and *Populus *proteomes were analyzed by GPCRHMM and QFC with the same software settings as those used for *Arabidopsis*.

Topology prediction was performed on the *Arabidopsis*, *Oryza*, and *Populus *proteomes by analyzing the complete proteomes with TMHMM version 2.0 [34], HMMTOP version 2.0 [36], and Phobius [37]. TMHMM2 was run using the 'one line per protein' option. HMMTOP2 was run in the advanced mode with the parameters: FASTA format, Single Sequence type, Reliable prediction type, text output, and the results in one line. Phobius was run in the Normal prediction mode with the short output format mode selected.

Signal peptide predictions were performed on the *Arabidopsis*, *Oryza*, and *Populus *proteomes using Phobius. Only a single run of Phobius is necessary to obtain signal peptide predictions and TM domain prediction as they are co-predicted. For those protein sequences identified as having a signal peptide by Phobius, the sequences were cleaved '*in silico*' and the predicted mature protein sequences were analyzed using TMHMM2 and HMMTOP2. Phobius was not utilized for TM domain prediction of the predicted mature protein sequences because the co-prediction analytical method of Phobius could lead to additional *in silico *cleavage of the mature proteins and consequent inaccurate TM domain prediction.

GPCR classification was performed using the GPCRsIdentifier executable program [50] and was applied to analyze the set of heptahelical proteins identified in our topological analyses of the *Arabidopsis*, *Oryza *and *Populus *proteomes. All of our candidate GPCRs from all three proteomes were assessed for coupling specificity using Pred-Couple 2 [51] and examined for the presence of domains catalogued in the Pfam database [52].

GPCRHMM, HMMTOP2, Phobius, Pred-Couple 2, and the Pfam queries were run using their respective public web servers, while the QFC algorithm was run locally on a LINUX cluster. The initial whole proteome analyses using TMHMM2 were kindly provided by Dr Jannick Bendtsen while subsequent analyses by TMHMM2 were performed over the internet. PRED-GPCR analyses of the *Arabidopsis *proteome were kindly provided by Dr Pantelis Bagos (University of Athens, Greece). The stand alone executable GPCRsIdentifier program was obtained from the author [50] and run locally. Results from the BTP method as published by Inoue *et al*. [80] were downloaded from the publisher's supplemental information website. The published BTP analysis was performed on the 2001 version of the *Arabidopsis *proteome and only protein sequences from their published results retaining an exact match to a protein sequence in the TAIR ATH1 version 7.0 were considered further.

It is notable that almost all of our whole proteome analyses were performed, or could have been performed, using the publicly available web servers in a reasonable amount of time with the exception of PRED-GPCR, which appears to time out while analyzing large batch submissions.

All the raw output files from the computational analyses were formatted, coded where appropriate, and used to create a relational database where the single unifying field between all tables for each respective proteome was the Locus identifier with splice variant information where available. BLASTClust (NCBI) was used to create the set of non-redundant proteins for each proteome with the percent identity and sequence length options set to 100% and the alignment length threshold enforced for all sequences. Redundant proteins were handled by using the lowest numerical identifier within a redundant protein set as a representative identifier. The data sets of corresponding splice variant or other protein redundancies within each proteome are available as Additional data files 12, 13, and 14.

### Identification of candidate GPCR homologs

*Arabidopsis*, *Oryza *and *Populus *protein sequences potentially orthologous to our *Arabidopsis *high ranking candidate GPCRs were identified using the BLOSUM62 scoring matrix and the BLAST algorithm implemented as a module in the BioEdit software package [89], with a cutoff value of e^-20^. Additional analyses performed to identify homologous sequences were performed using the public BLAST service at NCBI. Multiple sequence alignments were prepared in DAMBE [90] using ClustalW and the Blosum series protein matrix. Phylogenetic trees were reconstructed in MEGA4 [91] using the neighbor joining method with pairwise deletion of alignment gaps, Poisson correction for amino acid substitutions, and 1,000 bootstrap replicates.

### Protein-protein interaction assays

Coupling of the *Arabidopsis *heterotrimeric G-protein α subunit, GPA1, and proteins selected from the highest ranking pool of candidate GPCRs was experimentally investigated using the membrane-based split-ubiquitin system assay [68,82]. Split-ubiquitin system linker adapted gene specific primer pairs were designed to include a 5' translation initiation codon but not a 3' termination codon and were used to amplify the full length open reading frame cDNAs of the candidate GPCRs and GPA1. The cDNAs were cloned into the TOPO-BLUNT II vector (Invitrogen, Carlsbad, California, U.S.A), sequenced, and the inserts were recovered by restriction digestion and gel purification. The Nub_wt_, Nub_G _and Cub fusion constructs were created by homologous recombination following co-transformation of 50-100 ng of insert and 50-100 ng of linearized split-ubiquitin system vector into the haploid AP5 and AP4 yeast strains. AP4 transformants containing a Cub fusion construct were mated to AP5 transformants having one of the four Nub fusion constructs and then selected on SD minimal media.

Protein-protein interaction was assayed by patching diploid cultures to SD minimal media plates lacking His and Ade but containing either 0 μM, 200 μM, or 1 mM methionine and scored by visualization of yeast growth after 3-5 days. All experiments were independently replicated at least twice starting from the co-transformation stage.

## Abbreviations

7TMpR, 7TM putative receptor; BTP, binary topology pattern; Cub, carboxy-terminal half of ubiquitin; GCR, G-protein coupled receptor (from plants); GPCR, G-protein coupled receptor; HHP, heptahelical protein; HMM, hidden Markov model; MeSA, methyl salicylate; MLO, mildew resistance locus o; NubG, low affinity mutant of Nub_wt_; Nub_wt_, wild type amino-terminal half of ubiquitin; QFC, quasi-periodic feature classifier; RGS, regulator of G-protein signaling; TM, transmembrane; TOM, tobamovirus replication protein.

## Authors' contributions

SMA conceived of and supervised the study. TEG contributed study design and performed the computational and wet-bench analyses. SMA and TEG co-wrote the manuscript. JK contributed to both the analysis and manuscript preparation. All authors edited and approved the final manuscript.

## Additional data files

The following additional data are available with the online version of this paper. Additional data files 1, 2, 3 are tables containing bioinformatic characterizations of our second tier candidate G-protein coupled receptors from the *Arabidopsis*, *Oryza*, and *Populus *proteomes, respectively. Additional data file 4 contains a reconstructed phylogenetic tree of the Cand6/7 GPCR 'superfamily'. Additional data file 5 contains a multiple sequence alignment of Cand7 (At5g18520) and its closest homologs. Additional data files 6 and 7 contain tables listing results from our Pfam A and Pfam B database analyses, respectively. Additional data file 8 contains a table listing the *Arabidopsis *sequences removed from our analysis by our QFC ion channel filter. Additional data file 9 is a table containing the PredCouple 2 predicted coupling specificities of our candidate GPCRs from all three proteomes. Additional data file 10 is a table presenting our bioinformatic characterization of the *Arabidopsis *proteome (version 6) sequences predicted to be candidate GPCRs by PRED-GPCR. Additional data file 11 is a Venn diagram detailing the extent of overlap between candidate GPCRs predicted by our analysis and that of Moriyama *et al*. [49]. Additional data files 12, 13, and 14 are tables identifying protein redundancies within the *Arabidopsis*, *Oryza*, and *Populus *proteomes, respectively. Additional data files 12, 13, and 14 also contain the complete list of *Arabidopsis*, *Oryza*, and *Populus *protein sequence identifiers, including splice variant identifiers, used in this study.

## Supplementary Material

Additional data file 1 Bioinformatic characterization of our second tier candidate G-protein coupled receptors from the *Arabidopsis *proteome. Click here for file

Additional data file 2 Bioinformatic characterization of our second tier candidate G-protein coupled receptors from the *Oryza *proteome. Click here for file

Additional data file 3 Bioinformatic characterization of our second tier candidate G-protein coupled receptors from the *Populus *proteome. Click here for file

Additional data file 4 Reconstructed phylogenetic tree of the Cand6/7 GPCR 'superfamily'. Click here for file

Additional data file 5 Multiple sequence alignment of Cand7 (At5g18520) and its closest homologs. Click here for file

Additional data file 6 Results from our Pfam A database analysis. Click here for file

Additional data file 7 Results from our Pfam B database analysis. Click here for file

Additional data file 8 *Arabidopsis *sequences removed from our analysis by our QFC ion channel filter. Click here for file

Additional data file 9 PredCouple 2 predicted coupling specificities of our candidate GPCRs from all three proteomes. Click here for file

Additional data file 10 Bioinformatic characterization of the *Arabidopsis *proteome (version 6) sequences predicted to be candidate GPCRs by PRED-GPCR. Click here for file

Additional data file 11 Venn diagram detailing the extent of overlap between candidate GPCRs predicted by our analysis and that of Moriyama *et al*. [49]. Click here for file

Additional data file 12 Also listed are *Arabidopsis *protein sequence identifiers, including splice variant identifiers, used in this study Click here for file

Additional data file 13 Also listed are *Oryza *protein sequence identifiers, including splice variant identifiers, used in this study Click here for file

Additional data file 14 Also listed are *Populus *protein sequence identifiers, including splice variant identifiers, used in this study Click here for file
