# Supplementary material for: Whole proteome identification of plant candidate G-protein coupled receptors in Arabidopsis, rice, and poplar: computational prediction and in-vivo protein coupling
Source: Genome Biol. 2008 Jul 31;9(7):R120. doi: 10.1186/gb-2008-9-7-r120 (PMC2530877; doi:10.1186/gb-2008-9-7-r120)
Supplement: Additional data file 1 — Bioinformatic characterization of our second tier candidate G-protein coupled receptors from the Arabidopsis proteome. [file gb-2008-9-7-r120-S1.doc]

Additional Data File 1. **Characterization of our second tier *Arabidopsis* candidate G-protein coupled receptors.**

Candidates were chosen based on the criterion of positive identification by the QFC algorithm and a predicted topology of 7TM domains by at least two of the three topology prediction programs while also considering the presence of predicted signal peptides. Pcut-T and Pcut-H describe topology predictions of the mature proteins by TMHMM and HMMTOP, respectively, after in-silico cleavage at the signal peptide cleavage site predicted by Phobius. Loci in boldface are homologous to sequences identified in our table of high ranking *Arabidopsis* candidate GPCRs (Table 1). ^a^ Homologous to HHP2, HHP3 and HHP4. ^b^ Homologous to Cand9 and Cand10

| **Locus** | **QFC** | **TMHMM** | **HMMTOP** | **Phobius** | **Pcut-T** | **Pcut-H** |
| --- | --- | --- | --- | --- | --- | --- |
| At1g03070.1 | yes | 7 (in) | 7 (out) | 7 (out) |  |  |
| At1g10660.1 | yes | 7 (out) | 7 (out) | 7 (out) |  |  |
| **At1g11200.1^b^** | yes | 7 (out) | 7 (out) | 7 (out) |  |  |
| At1g11450.1 | yes | 7 (out) | 7 (out) | 7 (out) |  |  |
| At1g12750.1 | yes | 7 (in) | 7 (in) | 7 (in) |  |  |
| At1g16560.1 | yes | 8 (in) | 7 (out) | 7 (out) | 7 (out) | 7 (out) |
| At1g16560.4 | yes | 7 (out) | 7 (out) | 7 (out) |  |  |
| At1g21460.1 | yes | 7 (out) | 7 (out) | 7 (out) |  |  |
| At1g47640.1 | yes | 7 (out) | 7 (out) | 6 (in) |  |  |
| At1g49470.1 | yes | 7 (out) | 7 (out) | 7 (out) |  |  |
| At1g52580.1 | yes | 7 (out) | 7 (out) | 7 (in) |  |  |
| At1g55230.1 | yes | 7 (out) | 7 (out) | 7 (out) |  |  |
| At1g55240.1 | yes | 7 (out) | 6 (out) | 7 (out) |  |  |
| At1g60600.1 | yes | 8 (out) | 9 (out) | 7 (out) | 7 (in) | 8 (in) |
| At1g61800.1 | yes | 7 (in) | 7 (in) | 8 (in) |  |  |
| At1g62430.1 | yes | 7 (out) | 8 (out) | 7 (out) |  |  |
| At1g63110.2 | yes | 7 (in) | 7 (out) | 10 (in) |  |  |
| At1g63120.1 | yes | 7 (in) | 6 (out) | 7 (in) |  |  |
| At1g64150.1 | yes | 7 (in) | 7 (in) | 7 (in) |  |  |
| At1g66770.1 | yes | 6 (in) | 7 (out) | 7 (out) |  |  |
| At1g68820.1 | yes | 7 (in) | 7 (in) | 7 (out) | 5 (in) | 7 (in) |
| At1g71190.1 | yes | 6 (in) | 6 (in) | 7 (out) | 6 (in) | 7 (out) |
| At1g72590.1 | yes | 5 (in) | 7 (out) | 7 (out) |  |  |
| At1g75000.1 | yes | 6 (in) | 7 (out) | 7 (out) |  |  |
| At1g77860.1 | yes | 7 (in) | 7 (in) | 7 (in) |  |  |
| At2g16530.1 | yes | 5 (in) | 7 (out) | 7 (out) |  |  |
| At2g16530.2 | yes | 5 (in) | 7 (out) | 7 (out) |  |  |
| At2g17430.1 | yes | 8 (in) | 10 (in) | 7 (out) | 7 (out) | 9 (out) |
| At2g21080.1 | yes | 6 (out) | 7 (in) | 7 (in) |  |  |
| **At2g24150.1^a^** | yes | 7 (in) | 8 (out) | 7 (in) |  |  |
| At2g25810.1 | yes | 7 (in) | 7 (out) | 6 (in) |  |  |
| At2g29050.1 | yes | 7 (in) | 7 (in) | 7 (in) |  |  |
| At2g29050.2 | yes | 7 (in) | 7 (in) | 7 (in) |  |  |
| At2g31440.1 | yes | 7 (out) | 7 (out) | 7 (out) |  |  |
| At2g36830.1 | yes | 7 (in) | 7 (in) | 7 (out) |  |  |
| At2g37330.1 | yes | 7 (out) | 7 (out) | 5 (out) |  |  |
| At2g37450.1 | yes | 8 (in) | 8 (in) | 7 (out) | 7 (out) | 7 (out) |
| At2g39060.1 | yes | 7 (out) | 7 (out) | 7 (out) |  |  |
| At2g41610.1 | yes | 7 (out) | 7 (out) | 7 (out) |  |  |
| At2g46060.1 | yes | 5 (out) | 7 (out) | 7 (out) | 5 (out) | 7 (out) |
| At2g47115.1 | yes | 7 (out) | 7 (out) | 7 (out) |  |  |
| At3g01550.1 | yes | 7 (out) | 7 (out) | 7 (out) |  |  |
| At3g06100.1 | yes | 6 (out) | 7 (in) | 7 (out) |  |  |
| At3g06460.1 | yes | 7 (out) | 7 (out) | 7 (out) |  |  |
| At3g06470.1 | yes | 7 (out) | 7 (out) | 7 (out) |  |  |
| At3g14770.1 | yes | 7 (out) | 7 (out) | 7 (out) |  |  |
| At3g16240.1 | yes | 6 (out) | 7 (out) | 7 (out) |  |  |
| At3g16690.1 | yes | 7 (out) | 7 (out) | 7 (out) |  |  |
| At3g20870.1 | yes | 8 (out) | 8 (out) | 7 (out) | 7 (in) | 7 (in) |
| At3g25585.3 | yes | 7 (out) | 7 (out) | 7 (out) |  |  |
| At3g25805.1 | yes | 7 (in) | 7 (in) | 7 (in) |  |  |
| At3g27770.1 | yes | 7 (out) | 7 (out) | 7 (out) |  |  |
| At3g27770.2 | yes | 5 (out) | 7 (out) | 7 (out) |  |  |
| At3g28007.1 | yes | 7 (in) | 7 (out) | 7 (out) |  |  |
| At3g28070.2 | yes | 7 (out) | 7 (out) | 7 (out) |  |  |
| At3g28130.1 | yes | 7 (out) | 7 (out) | 7 (out) |  |  |
| At3g48740.1 | yes | 7 (in) | 7 (out) | 7 (out) |  |  |
| At3g53780.2 | yes | 7 (in) | 7 (in) | 7 (in) |  |  |
| At3g54730.1 | yes | 4 (out) | 7 (in) | 7 (in) |  |  |
| At3g59520.1 | yes | 6 (in) | 7 (out) | 7 (out) |  |  |
| At3g60590.3 | yes | 6 (in) | 7 (in) | 7 (out) |  |  |
| At3g63310.1 | yes | 7 (in) | 7 (out) | 7 (out) |  |  |
| At4g01430.2 | yes | 7 (in) | 7 (in) | 7 (out) |  |  |
| At4g01450.3 | yes | 7 (out) | 7 (in) | 6 (in) |  |  |
| At4g02690.1 | yes | 7 (in) | 7 (out) | 7 (out) |  |  |
| At4g10360.1 | yes | 7 (out) | 6 (in) | 7 (out) |  |  |
| At4g10850.1 | yes | 7 (out) | 7 (out) | 7 (out) |  |  |
| At4g12030.1 | yes | 7 (out) | 8 (in) | 7 (out) |  |  |
| At4g13590.1 | yes | 5 (in) | 7 (in) | 7 (in) |  |  |
| At4g14730.1 | yes | 7 (in) | 7 (in) | 7 (in) |  |  |
| At4g15290.1 | yes | 7 (out) | 7 (in) | 7 (out) |  |  |
| At4g15470.1 | yes | 7 (in) | 7 (in) | 7 (in) |  |  |
| At4g15920.1 | yes | 7 (out) | 7 (out) | 7 (out) |  |  |
| At4g17580.1 | yes | 6 (in) | 7 (out) | 7 (out) |  |  |
| At4g19950.1 | yes | 7 (out) | 6 (in) | 7 (out) |  |  |
| At4g22340.1 | yes | 7 (out) | 8 (out) | 7 (out) |  |  |
| At4g22340.2 | yes | 7 (out) | 8 (out) | 7 (out) |  |  |
| At4g22340.3 | yes | 7 (out) | 8 (out) | 7 (out) |  |  |
| At4g23010.1 | yes | 7 (out) | 10 (in) | 7 (out) |  |  |
| At4g23070.1 | yes | 7 (out) | 7 (out) | 7 (out) |  |  |
| At4g24250.1 | yes | 7 (out) | 7 (out) | 7 (out) |  |  |
| At4g25010.1 | yes | 7 (out) | 7 (out) | 7 (out) |  |  |
| At4g25750.1 | yes | 7 (out) | 8 (in) | 7 (out) |  |  |
| At4g35080.1 | yes | 5 (in) | 7 (in) | 7 (in) |  |  |
| At4g36830.1 | yes | 7 (out) | 7 (out) | 7 (out) |  |  |
| At4g36850.1 | yes | 5 (out) | 7 (out) | 7 (out) |  |  |
| **At4g37680.1^a^** | yes | 6 (in) | 7 (in) | 7 (in) |  |  |
| At5g07250.1 | yes | 7 (in) | 7 (in) | 7 (in) |  |  |
| At5g13170.1 | yes | 7 (out) | 7 (out) | 7 (out) |  |  |
| At5g19870.1 | yes | 7 (out) | 7 (out) | 7 (out) |  |  |
| **At5g20270.1^a^** | yes | 7 (in) | 7 (in) | 7 (in) |  |  |
| At5g23660.1 | yes | 7 (out) | 7 (out) | 7 (out) |  |  |
| At5g23990.1 | yes | 7 (out) | 7 (out) | 8 (in) |  |  |
| At5g38380.2 | yes | 6 (in) | 7 (out) | 7 (out) |  |  |
| At5g40260.1 | yes | 7 (out) | 7 (out) | 7 (out) |  |  |
| At5g40670.1 | yes | 7 (out) | 7 (out) | 7 (out) |  |  |
| At5g42420.2 | yes | 5 (in) | 7 (out) | 7 (out) |  |  |
| At5g44860.1 | yes | 7 (out) | 6 (in) | 7 (out) |  |  |
| At5g45105.1 | yes | 8 (in) | 8 (in) | 7 (out) | 7 (out) | 7 (out) |
| At5g47120.1 | yes | 7 (out) | 6 (in) | 7 (in) |  |  |
| At5g50375.1 | yes | 6 (in) | 7 (in) | 7 (in) |  |  |
| At5g50790.1 | yes | 7 (out) | 7 (out) | 7 (out) |  |  |
| At5g50800.1 | yes | 7 (out) | 7 (out) | 7 (out) |  |  |
| At5g53190.1 | yes | 7 (out) | 7 (out) | 7 (out) |  |  |
| At5g55380.1 | yes | 7 (in) | 7 (in) | 9 (in) |  |  |
| At5g58560.1 | yes | 7 (out) | 6 (out) | 7 (out) | 7 (out) | 6 (out) |
| At5g59500.1 | yes | 7 (in) | 7 (in) | 8 (in) |  |  |
| At5g62960.1 | yes | 7 (out) | 7 (out) | 7 (out) |  |  |
| At5g65000.2 | yes | 6 (in) | 8 (in) | 7 (out) | 6 (in) | 7 (out) |
| At5g65970.1 | yes | 8 (in) | 9 (out) | 7 (out) | 7 (out) | 9 (out) |
| AtCg00020.1 | yes | 7 (in) | 6 (out) | 7 (out) |  |  |
