# Supplementary material for: Whole proteome identification of plant candidate G-protein coupled receptors in Arabidopsis, rice, and poplar: computational prediction and in-vivo protein coupling
Source: Genome Biol. 2008 Jul 31;9(7):R120. doi: 10.1186/gb-2008-9-7-r120 (PMC2530877; doi:10.1186/gb-2008-9-7-r120)
Supplement: Additional data file 2 — Bioinformatic characterization of our second tier candidate G-protein coupled receptors from the Oryza proteome. [file gb-2008-9-7-r120-S2.doc]

Addition Data File 2. **Characterization of our second tier *Oryza* candidate G-protein coupled receptors***.*

Candidates were chosen based on the criterion of positive identification by the QFC algorithm and a predicted topology of 7TM domains by at least two of the three topology prediction programs while also considering the presence of predicted signal peptides. Pcut-T and Pcut-H describe topology predictions of the mature proteins by TMHMM and HMMTOP, respectively, after in-silico cleavage at the signal peptide cleavage site predicted by Phobius. Loci in boldface have homologs in the table describing our high ranking *Oryza* candidate GPCRs (Table 5). ^a^ Homologous to Os01g61970.1, Os02g40550.1, Os04g42960.1, and Os06g04130.1 ^b^ Homologous to Os02g45870.1, Os03g36790.1, Os07g01250.1

| **Locus** | **QFC** | **TMHMM** | **HMMTOP** | **Phobius** | **Pcut-T** | **Pcut-H** |
| --- | --- | --- | --- | --- | --- | --- |
| Os01g07310.2 | yes | 7 (out) | 7 (out) | 6 (in) |  |  |
| Os01g07660.1 | yes | 7 (out) | 7 (in) | 7 (out) |  |  |
| Os01g07700.1 | yes | 7 (out) | 7 (out) | 7 (out) |  |  |
| Os01g08260.2 | yes | 7 (out) | 7 (out) | 7 (out) |  |  |
| Os01g12130.1 | yes | 7 (out) | 7 (out) | 7 (out) |  |  |
| Os01g13130.1 | yes | 7 (out) | 7 (out) | 7 (out) |  |  |
| Os01g14730.1 | yes | 4 (out) | 6 (in) | 1 (out) | 7 (in) | 7 (in) |
| Os01g23070.1 | yes | 7 (in) | 9 (out) | 7 (out) |  |  |
| Os01g25189.1 | yes | 7 (out) | 9 (out) | 7 (out) | 7 (out) | 9 (out) |
| Os01g31870.3 | yes | 7 (out) | 7 (in) | 7 (in) |  |  |
| Os01g36070.1 | yes | 7 (out) | 7 (out) | 7 (out) | 7 (out) | 7 (out) |
| Os01g42090.1 | yes | 7 (out) | 7 (out) | 7 (out) |  |  |
| Os01g42110.1 | yes | 7 (out) | 7 (out) | 7 (out) |  |  |
| Os01g42340.1 | yes | 7 (out) | 6 (out) | 7 (out) |  |  |
| Os01g48640.1 | yes | 8 (in) | 8 (in) | 7 (out) | 7 (out) | 8 (in) |
| Os01g50460.1 | yes | 7 (out) | 7 (out) | 7 (out) |  |  |
| Os01g55360.1 | yes | 7 (out) | 8 (out) | 7 (out) |  |  |
| Os01g55360.4 | yes | 7 (out) | 7 (out) | 7 (out) |  |  |
| Os01g57710.2 | yes | 7 (in) | 7 (in) | 6 (out) | 7 (in) | 7 (in) |
| Os01g58620.1 | yes | 7 (out) | 6 (in) | 7 (out) |  |  |
| Os01g60120.1 | yes | 7 (out) | 6 (in) | 7 (out) |  |  |
| Os01g61010.2 | yes | 7 (out) | 8 (in) | 7 (out) |  |  |
| **Os01g61960.1^a^** | yes | 7 (out) | 7 (out) | 7 (out) |  |  |
| Os01g65150.1 | yes | 7 (out) | 12 (in) | 7 (out) |  |  |
| Os01g65200.1 | yes | 7 (in) | 7 (in) | 6 (in) |  |  |
| Os01g65880.1 | yes | 6 (in) | 7 (out) | 7 (out) |  |  |
| Os01g67040.1 | yes | 6 (in) | 7 (out) | 7 (out) |  |  |
| Os01g69010.1 | yes | 7 (out) | 8 (in) | 7 (out) |  |  |
| Os01g72210.1 | yes | 7 (out) | 11 (out) | 7 (out) |  |  |
| Os01g74450.1 | yes | 7 (out) | 6 (in) | 7 (out) |  |  |
| Os02g02750.2 | yes | 8 (in) | 7 (out) | 7 (out) | 7 (out) | 7 (out) |
| Os02g03280.1 | yes | 7 (in) | 6 (in) | 7 (in) |  |  |
| Os02g03790.1 | yes | 7 (out) | 7 (out) | 7 (out) | 7 (out) | 7 (out) |
| Os02g17280.1 | yes | 7 (out) | 7 (out) | 7 (out) |  |  |
| Os02g19470.1 | yes | 7 (out) | 7 (out) | 7 (out) |  |  |
| Os02g19680.1 | yes | 7 (out) | 7 (out) | 9 (out) |  |  |
| Os02g19820.1 | yes | 7 (out) | 7 (out) | 7 (out) |  |  |
| Os02g29510.2 | yes | 7 (out) | 7 (out) | 7 (out) |  |  |
| Os02g30910.1 | yes | 7 (out) | 7 (out) | 7 (out) |  |  |
| Os02g32930.1 | yes | 7 (out) | 10 (in) | 7 (out) |  |  |
| Os02g35830.1 | yes | 7 (in) | 8 (in) | 7 (in) |  |  |
| Os02g39200.2 | yes | 7 (in) | 7 (out) | 9 (out) |  |  |
| Os02g41780.1 | yes | 8 (in) | 8 (in) | 9 (out) | 7 (out) | 7 (out) |
| Os02g43410.1 | yes | 7 (out) | 7 (out) | 8 (in) |  |  |
| Os02g43410.2 | yes | 7 (out) | 7 (out) | 8 (in) |  |  |
| Os02g44910.1 | yes | 7 (out) | 7 (out) | 5 (out) |  |  |
| Os02g57570.1 | yes | 6 (out) | 7 (in) | 7 (in) |  |  |
| Os03g02530.1 | yes | 7 (out) | 7 (in) | 7 (in) |  |  |
| **Os03g02850.1^b^** | yes | 7 (out) | 7 (out) | 7 (out) |  |  |
| **Os03g02850.2^b^** | yes | 7 (out) | 7 (out) | 7 (out) |  |  |
| Os03g03590.1 | yes | 6 (in) | 7 (out) | 7 (out) |  |  |
| Os03g03590.2 | yes | 6 (in) | 7 (out) | 7 (out) |  |  |
| Os03g13040.1 | yes | 6 (in) | 7 (in) | 7 (in) |  |  |
| Os03g14880.1 | yes | 6 (in) | 7 (out) | 7 (out) |  |  |
| Os03g21690.1 | yes | 7 (out) | 8 (in) | 7 (out) | 7 (out) | 7 (out) |
| Os03g22590.1 | yes | 7 (out) | 7 (out) | 7 (out) |  |  |
| Os03g40700.1 | yes | 7 (in) | 8 (in) | 7 (in) |  |  |
| Os03g49480.1 | yes | 7 (out) | 7 (out) | 7 (out) |  |  |
| Os03g49900.1 | yes | 7 (out) | 5 (in) | 7 (out) |  |  |
| Os03g53400.1 | yes | 7 (in) | 7 (in) | 7 (in) |  |  |
| Os03g58140.1 | yes | 7 (out) | 7 (in) | 7 (in) |  |  |
| Os03g58150.1 | yes | 7 (in) | 7 (in) | 7 (in) |  |  |
| Os03g61210.1 | yes | 6 (out) | 7 (in) | 7 (out) |  |  |
| Os04g16770.1 | yes | 7 (in) | 6 (out) | 7 (out) |  |  |
| Os04g32050.2 | yes | 7 (in) | 9 (in) | 7 (in) |  |  |
| Os04g36680.1 | yes | 6 (out) | 7 (out) | 7 (out) |  |  |
| Os04g38850.1 | yes | 6 (in) | 7 (in) | 7 (out) | 6 (in) | 7 (in) |
| Os04g38860.1 | yes | 7 (in) | 10 (in) | 7 (in) |  |  |
| Os04g48130.1 | yes | 7 (out) | 7 (in) | 7 (in) |  |  |
| Os04g51970.1 | yes | 7 (out) | 7 (out) | 7 (out) | 6 (in) | 7 (out) |
| Os05g02750.1 | yes | 7 (out) | 7 (out) | 5 (out) | 7 (out) | 7 (out) |
| Os05g12320.1 | yes | 7 (out) | 7 (out) | 7 (out) |  |  |
| Os05g13330.1 | yes | 8 (in) | 8 (in) | 7 (out) | 7 (out) | 7 (out) |
| Os05g13330.2 | yes | 7 (out) | 7 (out) | 7 (out) |  |  |
| Os05g25890.1 | yes | 7 (out) | 6 (out) | 7 (out) |  |  |
| Os05g28950.1 | yes | 7 (out) | 7 (in) | 8 (out) |  |  |
| Os05g30150.1 | yes | 7 (in) | 7 (in) | 7 (in) |  |  |
| Os05g33360.1 | yes | 7 (in) | 7 (in) | 7 (in) |  |  |
| Os05g35140.1 | yes | 6 (in) | 7 (out) | 7 (out) |  |  |
| Os05g51090.1 | yes | 7 (in) | 7 (out) | 7 (out) |  |  |
| Os06g02370.2 | yes | 7 (out) | 8 (in) | 7 (in) |  |  |
| Os06g03820.1 | yes | 7 (out) | 7 (out) | 7 (out) |  |  |
| Os06g05980.2 | yes | 7 (in) | 7 (out) | 7 (out) |  |  |
| Os06g22600.1 | yes | 7 (in) | 7 (out) | 6 (in) |  |  |
| Os06g35930.1 | yes | 6 (in) | 7 (out) | 7 (out) |  |  |
| Os06g42850.1 | yes | 6 (in) | 7 (in) | 7 (in) |  |  |
| Os06g43620.1 | yes | 7 (in) | 9 (in) | 7 (in) |  |  |
| Os06g44250.1 | yes | 7 (in) | 7 (in) | 8 (out) |  |  |
| Os06g44840.1 | yes | 7 (out) | 7 (in) | 8 (out) |  |  |
| Os06g46820.2 | yes | 7 (in) | 7 (in) | 7 (in) |  |  |
| Os07g08060.1 | yes | 7 (in) | 7 (in) | 7 (in) |  |  |
| Os07g08070.1 | yes | 7 (in) | 7 (in) | 7 (in) |  |  |
| Os07g30100.2 | yes | 4 (in) | 5 (in) | 7 (out) | 3 (out) | 7 (out) |
| Os07g31140.1 | yes | 5 (out) | 7 (out) | 7 (out) |  |  |
| Os07g33780.1 | yes | 6 (in) | 7 (out) | 7 (out) |  |  |
| Os07g42310.1 | yes | 8 (in) | 10 (in) | 7 (out) | 7 (out) | 7 (out) |
| Os07g46430.2 | yes | 5 (out) | 7 (out) | 7 (out) |  |  |
| Os08g01610.1 | yes | 7 (out) | 8 (in) | 7 (out) |  |  |
| Os08g03430.1 | yes | 8 (in) | 8 (in) | 7 (out) | 7 (out) | 8 (in) |
| Os08g25830.1 | yes | 7 (out) | 7 (out) | 7 (out) |  |  |
| Os08g36030.1 | yes | 7 (out) | 7 (out) | 7 (out) |  |  |
| Os08g36040.1 | yes | 3 (out) | 7 (out) | 7 (out) |  |  |
| Os08g42350.1 | yes | 7 (out) | 7 (out) | 7 (out) |  |  |
| Os08g43320.1 | yes | 7 (in) | 7 (in) | 7 (in) |  |  |
| Os09g12600.1 | yes | 7 (out) | 7 (in) | 9 (out) | 7 (out) | 7 (in) |
| Os09g26830.1 | yes | 8 (in) | 8 (in) | 7 (out) | 7 (out) | 7 (out) |
| Os09g27250.1 | yes | 5 (out) | 7 (out) | 7 (out) |  |  |
| Os09g29210.2 | yes | 7 (in) | 7 (in) | 7 (in) |  |  |
| Os09g30446.1 | yes | 7 (out) | 8 (in) | 7 (out) |  |  |
| Os09g35730.1 | yes | 7 (in) | 7 (in) | 7 (out) |  |  |
| Os09g38690.4 | yes | 7 (out) | 7 (out) | 7 (out) |  |  |
| Os09g39220.1 | yes | 7 (out) | 7 (in) | 7 (out) | 7 (out) | 7 (in) |
| Os10g12750.1 | yes | 6 (in) | 7 (out) | 7 (out) |  |  |
| Os10g14920.5 | yes | 7 (out) | 7 (out) | 7 (out) |  |  |
| Os10g21200.1 | yes | 7 (in) | 8 (out) | 7 (out) |  |  |
| Os10g30910.1 | yes | 6 (in) | 7 (in) | 7 (out) | 5 (out) | 7 (out) |
| Os10g38030.1 | yes | 8 (in) | 8 (in) | 7 (out) | 6 (in) | 7 (out) |
| **Os10g39220.1^b^** | yes | 6 (in) | 8 (in) | 7 (out) | 7 (out) | 7 (out) |
| Os11g01410.1 | yes | 7 (out) | 8 (out) | 7 (out) |  |  |
| Os11g04140.1 | yes | 7 (in) | 7 (in) | 7 (out) |  |  |
| Os11g06330.1 | yes | 8 (in) | 7 (out) | 7 (out) |  |  |
| Os11g19700.1 | yes | 6 (in) | 7 (in) | 7 (in) |  |  |
| Os11g31190.1 | yes | 7 (out) | 7 (out) | 7 (out) |  |  |
| Os11g34180.1 | yes | 5 (in) | 7 (in) | 7 (in) |  |  |
| Os11g34360.1 | yes | 8 (in) | 8 (in) | 7 (out) | 7 (out) | 7 (out) |
| Os11g37200.1 | yes | 7 (in) | 7 (in) | 7 (in) |  |  |
| Os11g47840.1 | yes | 7 (in) | 7 (in) | 7 (in) |  |  |
| Os12g03200.1 | yes | 4 (in) | 7 (in) | 7 (in) |  |  |
| Os12g03230.1 | yes | 7 (out) | 7 (out) | 7 (out) |  |  |
| Os12g03950.1 | yes | 7 (out) | 7 (out) | 7 (out) |  |  |
| Os12g18110.1 | yes | 7 (out) | 7 (out) | 7 (out) |  |  |
| Os12g18110.2 | yes | 7 (out) | 7 (out) | 7 (out) |  |  |
| Os12g29220.1 | yes | 7 (out) | 7 (out) | 7 (out) |  |  |
| Os12g31890.1 | yes | 7 (out) | 8 (out) | 7 (out) |  |  |
| Os12g32640.1 | yes | 7 (out) | 7 (out) | 7 (out) |  |  |
| Os12g38810.1 | yes | 6 (in) | 6 (in) | 7 (out) | 6 (in) | 7 (out) |
| Os12g41840.1 | yes | 7 (in) | 7 (in) | 7 (out) | 6 (out) | 7 (in) |
| Os12g43890.1 | yes | 6 (in) | 7 (out) | 7 (out) |  |  |
