# Supplementary material for: Whole proteome identification of plant candidate G-protein coupled receptors in Arabidopsis, rice, and poplar: computational prediction and in-vivo protein coupling
Source: Genome Biol. 2008 Jul 31;9(7):R120. doi: 10.1186/gb-2008-9-7-r120 (PMC2530877; doi:10.1186/gb-2008-9-7-r120)
Supplement: Additional data file 3 — Bioinformatic characterization of our second tier candidate G-protein coupled receptors from the Populus proteome. [file gb-2008-9-7-r120-S3.doc]

Additional Data File 3. **Characterization of our second tier *Populus* candidate G-protein coupled receptors.**

Candidates were chosen based on the criterion of positive identification by the QFC algorithm and a predicted topology of 7TM domains by at least two of the three topology prediction programs while also considering the presence of predicted signal peptides. Pcut-T and Pcut-H describe topology predictions of the mature proteins by TMHMM and HMMTOP, respectively, after in-silico cleavage at the signal peptide cleavage site predicted by Phobius. The locus in boldface has three homologs in the table describing our highest ranking *Populus* candidate GPCRs (Table 6). ^a^ Homologous to Pop254437, Pop273474, and Pop762585

| **Locus** | **QFC** | **TMHMM** | **HMMTOP** | **Phobius** | **Pcut-T** | **Pcut-H** |
| --- | --- | --- | --- | --- | --- | --- |
| Pop172832 | yes | 7 (in) | 9 (in) | 7 (out) |  |  |
| Pop173036 | yes | 7 (out) | 9 (out) | 7 (out) |  |  |
| Pop173892 | yes | 7 (out) | 6 (in) | 7 (out) |  |  |
| Pop175785 | yes | 7 (out) | 6 (in) | 7 (out) |  |  |
| Pop177980 | yes | 7 (out) | 7 (out) | 7 (out) |  |  |
| Pop177987 | yes | 7 (out) | 7 (out) | 7 (out) |  |  |
| Pop179024 | yes | 5 (in) | 7 (in) | 7 (in) |  |  |
| Pop179233 | yes | 7 (out) | 7 (out) | 7 (out) |  |  |
| Pop196703 | yes | 7 (in) | 7 (in) | 7 (in) |  |  |
| Pop200605 | yes | 8 (in) | 8 (out) | 7 (out) | 7 (out) | 7 (in) |
| Pop201535 | yes | 7 (out) | 7 (out) | 6 (in) |  |  |
| Pop204757 | yes | 7 (out) | 7 (out) | 7 (out) |  |  |
| Pop206779 | yes | 7 (out) | 9 (out) | 7 (out) |  |  |
| Pop206792 | yes | 7 (out) | 8 (in) | 7 (out) |  |  |
| Pop208168 | yes | 7 (out) | 9 (out) | 7 (out) |  |  |
| Pop215806 | yes | 7 (in) | 8 (out) | 7 (in) |  |  |
| Pop215987 | yes | 7 (out) | 9 (out) | 7 (out) |  |  |
| Pop216767 | yes | 7 (out) | 7 (in) | 8 (in) |  |  |
| Pop218133 | yes | 7 (out) | 9 (out) | 7 (out) |  |  |
| Pop218443 | yes | 7 (out) | 9 (out) | 7 (out) |  |  |
| Pop219003 | yes | 4 (in) | 7 (out) | 7 (out) |  |  |
| Pop225580 | yes | 7 (out) | 7 (in) | 7 (in) |  |  |
| Pop225611 | yes | 7 (in) | 9 (in) | 7 (out) |  |  |
| Pop225724 | yes | 7 (out) | 9 (out) | 7 (out) |  |  |
| Pop227902 | yes | 7 (out) | 9 (out) | 7 (out) |  |  |
| Pop230624 | yes | 7 (out) | 7 (out) | 7 (out) |  |  |
| Pop235962 | yes | 7 (in) | 7 (out) | 7 (out) |  |  |
| Pop240504 | yes | 7 (out) | 7 (out) | 7 (out) |  |  |
| Pop242137 | yes | 7 (out) | 7 (in) | 8 (in) |  |  |
| Pop246659 | yes | 7 (out) | 6 (in) | 7 (out) |  |  |
| Pop252021 | yes | 7 (in) | 7 (out) | 7 (out) |  |  |
| Pop252572 | yes | 7 (in) | 8 (in) | 7 (in) |  |  |
| Pop252617 | yes | 7 (out) | 7 (in) | 7 (in) |  |  |
| Pop252864 | yes | 7 (out) | 7 (out) | 7 (out) |  |  |
| Pop254201 | yes | 6 (in) | 7 (in) | 7 (out) |  |  |
| Pop256648 | yes | 7 (out) | 7 (out) | 5 (out) |  |  |
| Pop256812 | yes | 7 (out) | 9 (out) | 7 (out) |  |  |
| Pop257635 | yes | 7 (out) | 7 (out) | 7 (out) | 6 (in) | 7 (out) |
| Pop259300 | yes | 7 (in) | 7 (in) | 8 (in) |  |  |
| Pop259524 | yes | 7 (in) | 7 (in) | 7 (in) |  |  |
| Pop261855 | yes | 7 (out) | 7 (out) | 7 (out) |  |  |
| Pop262559 | yes | 7 (in) | 7 (in) | 7 (in) |  |  |
| Pop263939 | yes | 7 (in) | 7 (out) | 7 (out) | 5 (out) | 7 (out) |
| Pop265277 | yes | 4 (out) | 7 (out) | 7 (out) |  |  |
| Pop267247 | yes | 7 (in) | 7 (in) | 9 (out) |  |  |
| Pop267303 | yes | 4 (out) | 7 (out) | 7 (out) |  |  |
| Pop267622 | yes | 4 (out) | 7 (out) | 7 (out) |  |  |
| Pop268014 | yes | 7 (out) | 7 (in) | 7 (out) |  |  |
| Pop268529 | yes | 4 (out) | 7 (out) | 7 (out) |  |  |
| Pop269497 | yes | 6 (in) | 7 (out) | 7 (out) |  |  |
| Pop269792 | yes | 4 (out) | 7 (out) | 7 (out) |  |  |
| Pop270465 | yes | 7 (out) | 8 (in) | 7 (out) | 7 (out) | 8 (in) |
| Pop271644 | yes | 7 (in) | 7 (in) | 7 (in) |  |  |
| Pop272750 | yes | 7 (in) | 7 (out) | 7 (in) |  |  |
| Pop273366 | yes | 7 (in) | 7 (in) | 7 (in) |  |  |
| Pop274865 | yes | 7 (out) | 8 (in) | 7 (out) |  |  |
| Pop275921 | yes | 4 (out) | 7 (out) | 7 (out) |  |  |
| Pop276316 | yes | 7 (out) | 7 (out) | 7 (out) |  |  |
| Pop276782 | yes | 7 (out) | 8 (out) | 7 (out) |  |  |
| Pop277455 | yes | 7 (out) | 7 (out) | 7 (out) |  |  |
| Pop281777 | yes | 4 (in) | 8 (in) | 7 (out) | 3 (out) | 7 (out) |
| Pop282629 | yes | 7 (in) | 7 (in) | 7 (in) |  |  |
| Pop283981 | yes | 7 (out) | 7 (out) | 7 (out) |  |  |
| Pop284619 | yes | 7 (in) | 7 (in) | 7 (in) |  |  |
| Pop284734 | yes | 6 (out) | 7 (in) | 7 (in) |  |  |
| Pop286007 | yes | 7 (in) | 7 (out) | 4 (in) |  |  |
| Pop286694 | yes | 7 (out) | 7 (out) | 7 (out) |  |  |
| Pop288106 | yes | 5 (out) | 7 (out) | 7 (out) |  |  |
| Pop289249 | yes | 7 (in) | 10 (in) | 7 (in) |  |  |
| Pop289951 | yes | 7 (out) | 7 (out) | 7 (out) |  |  |
| Pop412008 | yes | 7 (out) | 8 (in) | 7 (out) |  |  |
| Pop412037 | yes | 7 (out) | 7 (out) | 7 (out) |  |  |
| Pop414177 | yes | 7 (out) | 7 (out) | 7 (out) |  |  |
| Pop414178 | yes | 7 (out) | 7 (out) | 7 (out) |  |  |
| **Pop414302^a^** | yes | 7 (out) | 7 (out) | 7 (out) |  |  |
| Pop414904 | yes | 7 (out) | 7 (out) | 7 (out) |  |  |
| Pop415791 | yes | 7 (in) | 7 (in) | 7 (in) |  |  |
| Pop416545 | yes | 7 (out) | 7 (in) | 9 (out) |  |  |
| Pop417284 | yes | 4 (in) | 7 (out) | 7 (out) |  |  |
| Pop417676 | yes | 9 (in) | 8 (out) | 8 (out) | 7 (in) | 7 (in) |
| Pop419541 | yes | 7 (out) | 8 (out) | 7 (out) |  |  |
| Pop421006 | yes | 5 (in) | 7 (out) | 7 (out) |  |  |
| Pop421765 | yes | 7 (out) | 7 (out) | 7 (out) |  |  |
| Pop421875 | yes | 7 (in) | 6 (out) | 7 (out) |  |  |
| Pop422093 | yes | 7 (out) | 7 (out) | 7 (out) |  |  |
| Pop423394 | yes | 7 (out) | 7 (out) | 7 (out) |  |  |
| Pop423491 | yes | 7 (out) | 7 (out) | 6 (out) |  |  |
| Pop548890 | yes | 7 (out) | 7 (out) | 7 (out) |  |  |
| Pop549212 | yes | 6 (out) | 6 (in) | 7 (out) | 7 (in) | 6 (in) |
| Pop551452 | yes | 7 (out) | 7 (out) | 8 (in) |  |  |
| Pop552585 | yes | 7 (out) | 7 (out) | 7 (out) |  |  |
| Pop553107 | yes | 7 (out) | 5 (out) | 7 (out) |  |  |
| Pop555238 | yes | 7 (out) | 7 (out) | 7 (out) |  |  |
| Pop555239 | yes | 7 (out) | 7 (out) | 7 (out) |  |  |
| Pop555701 | yes | 6 (out) | 7 (in) | 7 (out) |  |  |
| Pop557139 | yes | 7 (out) | 7 (out) | 7 (out) |  |  |
| Pop558261 | yes | 7 (out) | 9 (out) | 7 (out) |  |  |
| Pop559596 | yes | 7 (in) | 7 (in) | 7 (in) |  |  |
| Pop560115 | yes | 5 (in) | 7 (in) | 7 (in) |  |  |
| Pop561531 | yes | 7 (out) | 7 (out) | 5 (out) |  |  |
| Pop561759 | yes | 7 (out) | 7 (out) | 7 (out) |  |  |
| Pop562220 | yes | 7 (out) | 7 (out) | 7 (out) |  |  |
| Pop562882 | yes | 8 (in) | 7 (out) | 7 (out) | 7 (out) | 7 (out) |
| Pop564874 | yes | 7 (in) | 7 (in) | 7 (in) |  |  |
| Pop566501 | yes | 7 (out) | 6 (in) | 7 (out) |  |  |
| Pop567196 | yes | 6 (in) | 7 (in) | 7 (out) |  |  |
| Pop570518 | yes | 8 (in) | 8 (in) | 7 (out) | 7 (out) | 7 (out) |
| Pop570771 | yes | 7 (in) | 7 (out) | 7 (out) |  |  |
| Pop571983 | yes | 7 (in) | 6 (out) | 7 (in) |  |  |
| Pop575407 | yes | 7 (out) | 7 (out) | 7 (out) |  |  |
| Pop575408 | yes | 7 (in) | 7 (out) | 7 (out) |  |  |
| Pop575753 | yes | 6 (in) | 8 (in) | 7 (out) | 6 (in) | 7 (out) |
| Pop575848 | yes | 4 (out) | 7 (out) | 7 (out) |  |  |
| Pop576416 | yes | 7 (out) | 9 (out) | 7 (out) |  |  |
| Pop577709 | yes | 7 (in) | 7 (in) | 8 (in) |  |  |
| Pop579599 | yes | 7 (out) | 6 (in) | 7 (out) |  |  |
| Pop580803 | yes | 7 (out) | 7 (out) | 7 (out) |  |  |
| Pop581554 | yes | 6 (in) | 7 (out) | 7 (out) |  |  |
| Pop581835 | yes | 6 (in) | 7 (out) | 7 (out) |  |  |
| Pop581977 | yes | 7 (out) | 7 (out) | 7 (out) |  |  |
| Pop585035 | yes | 7 (out) | 9 (out) | 7 (out) |  |  |
| Pop587247 | yes | 7 (out) | 7 (out) | 7 (out) | 7 (out) | 7 (out) |
| Pop587453 | yes | 7 (out) | 7 (out) | 7 (out) |  |  |
| Pop589141 | yes | 8 (out) | 7 (in) | 7 (out) | 7 (in) | 7 (in) |
| Pop589144 | yes | 7 (out) | 7 (out) | 7 (out) |  |  |
| Pop590393 | yes | 7 (out) | 7 (out) | 7 (out) |  |  |
| Pop590580 | yes | 7 (out) | 7 (out) | 7 (out) |  |  |
| Pop592088 | yes | 7 (in) | 7 (out) | 7 (out) |  |  |
| Pop593823 | yes | 6 (out) | 7 (out) | 7 (out) |  |  |
| Pop594744 | yes | 7 (out) | 9 (out) | 7 (out) |  |  |
| Pop596138 | yes | 7 (out) | 7 (out) | 7 (out) |  |  |
| Pop597309 | yes | 6 (in) | 8 (in) | 7 (out) | 5 (out) | 7 (out) |
| Pop645978 | yes | 7 (in) | 7 (out) | 7 (out) |  |  |
| Pop654478 | yes | 7 (out) | 7 (out) | 7 (out) |  |  |
| Pop673369 | yes | 7 (out) | 7 (out) | 7 (out) |  |  |
| Pop680198 | yes | 7 (out) | 7 (out) | 6 (in) |  |  |
| Pop706133 | yes | 7 (in) | 7 (in) | 7 (in) |  |  |
| Pop706794 | yes | 7 (in) | 7 (out) | 7 (out) |  |  |
| Pop708621 | yes | 6 (in) | 7 (out) | 7 (out) |  |  |
| Pop710099 | yes | 7 (in) | 7 (out) | 7 (out) |  |  |
| Pop712123 | yes | 7 (out) | 7 (out) | 7 (out) |  |  |
| Pop720911 | yes | 7 (in) | 8 (out) | 7 (out) |  |  |
| Pop725778 | yes | 7 (in) | 7 (in) | 7 (in) |  |  |
| Pop729499 | yes | 6 (in) | 7 (out) | 7 (out) |  |  |
| Pop731487 | yes | 5 (out) | 7 (out) | 7 (out) |  |  |
| Pop744336 | yes | 7 (out) | 7 (in) | 7 (out) |  |  |
| Pop746292 | yes | 7 (out) | 9 (out) | 7 (out) |  |  |
| Pop754497 | yes | 7 (out) | 7 (out) | 7 (out) |  |  |
| Pop755787 | yes | 7 (out) | 7 (out) | 8 (out) |  |  |
| Pop759781 | yes | 7 (out) | 6 (in) | 7 (out) |  |  |
| Pop761806 | yes | 7 (in) | 6 (out) | 7 (out) |  |  |
| Pop764671 | yes | 6 (in) | 7 (out) | 7 (out) |  |  |
| Pop767334 | yes | 7 (out) | 7 (out) | 7 (out) |  |  |
| Pop769002 | yes | 7 (out) | 7 (out) | 7 (out) |  |  |
| Pop769737 | yes | 5 (out) | 7 (out) | 7 (out) |  |  |
| Pop775798 | yes | 7 (in) | 5 (in) | 7 (in) |  |  |
| Pop776100 | yes | 7 (out) | 7 (out) | 7 (out) |  |  |
| Pop776330 | yes | 7 (out) | 7 (out) | 7 (out) |  |  |
| Pop776383 | yes | 5 (out) | 7 (out) | 7 (out) |  |  |
| Pop776387 | yes | 7 (out) | 6 (out) | 7 (out) |  |  |
| Pop778684 | yes | 6 (in) | 7 (out) | 7 (out) |  |  |
| Pop796067 | yes | 6 (in) | 7 (in) | 7 (in) |  |  |
| Pop796925 | yes | 7 (out) | 8 (in) | 7 (out) |  |  |
| Pop797856 | yes | 7 (out) | 7 (out) | 7 (out) |  |  |
| Pop797910 | yes | 7 (out) | 6 (in) | 7 (out) |  |  |
| Pop799475 | yes | 7 (in) | 8 (in) | 7 (out) | 5 (in) | 7 (out) |
| Pop814068 | yes | 7 (in) | 7 (in) | 7 (in) |  |  |
| Pop815105 | yes | 7 (in) | 6 (out) | 7 (in) |  |  |
| Pop816235 | yes | 7 (out) | 7 (out) | 7 (out) |  |  |
| Pop816796 | yes | 7 (out) | 7 (in) | 8 (in) |  |  |
| Pop820600 | yes | 6 (out) | 7 (in) | 7 (in) |  |  |
| Pop821124 | yes | 7 (out) | 6 (in) | 7 (out) |  |  |
| Pop824920 | yes | 7 (in) | 6 (out) | 7 (in) |  |  |
| Pop826764 | yes | 7 (out) | 7 (out) | 6 (in) |  |  |
| Pop827617 | yes | 7 (out) | 7 (out) | 7 (out) |  |  |
| Pop829403 | yes | 7 (out) | 9 (out) | 7 (out) |  |  |
| Pop830825 | yes | 7 (in) | 7 (in) | 7 (in) |  |  |
| Pop831071 | yes | 5 (in) | 7 (out) | 7 (out) |  |  |
| Pop831600 | yes | 7 (in) | 7 (out) | 7 (out) |  |  |
| Pop835343 | yes | 6 (in) | 9 (in) | 8 (out) | 7 (out) | 7 (in) |
| Pop836846 | yes | 7 (out) | 8 (out) | 7 (out) |  |  |
| Pop837467 | yes | 7 (out) | 8 (out) | 7 (out) |  |  |
