# Supplementary material for: Whole proteome identification of plant candidate G-protein coupled receptors in Arabidopsis, rice, and poplar: computational prediction and in-vivo protein coupling
Source: Genome Biol. 2008 Jul 31;9(7):R120. doi: 10.1186/gb-2008-9-7-r120 (PMC2530877; doi:10.1186/gb-2008-9-7-r120)
Supplement: Additional data file 4 — Reconstructed phylogenetic tree of the Cand6/7 GPCR 'superfamily'. [file gb-2008-9-7-r120-S4.doc]

Additional Data File 4. **Molecular evolutionary analyses of the Cand6/7 candidate GPCR “superfamily”.** All high ranking candidate GPCRs from the *Arabidopsis*, *Oryza*, and *Populus* proteomes were used as queries for BLAST analyses (e^-20^ threshold) and the resultant sequences were subjected to cluster analysis. The Cand7 containing cluster was aligned using ClustalX, phylogeny was estimated using the Neighbor Joining method with 1000 bootstrap replicates, and the tree was rooted on the midpoint using At1g10980.1. The filled upward pointing triangle indicates Cand7 which we show physically interacts with GPA1. Sequences identified by our bioinformatic pipeline as candidate GPCRs are indicated with unfilled triangles, with upward pointing triangles indicating those found within our high ranking candidate sets and downward pointing triangles indicating those present in the second tier. *Arabidopsis* sequences that would have been predicted to be candidate GPCRs had we not utilized the QFC ion channel filter are marked with an asterisk, and the two close Cand7 *Arabidopsis* homologs that have at least a “2/3” heptahelical topology prediction and were directly predicted to be GPCRs by GPCRHMM, but not by the QFC algorithm, are shown with double asterisks.
