# Supplementary material for: Whole proteome identification of plant candidate G-protein coupled receptors in Arabidopsis, rice, and poplar: computational prediction and in-vivo protein coupling
Source: Genome Biol. 2008 Jul 31;9(7):R120. doi: 10.1186/gb-2008-9-7-r120 (PMC2530877; doi:10.1186/gb-2008-9-7-r120)
Supplement: Additional data file 5 — Multiple sequence alignment of Cand7 (At5g18520) and its closest homologs. [file gb-2008-9-7-r120-S5.rtf]

Additional Data File 5. Multiple sequence alignment of Cand7 (At5g18520) and its closest homologs. Sequences were identified by BLAST analyses (e-20 cutoff) of the Arabidopsis, Oryza, and Populus proteomes using Cand7 as the query. The closest mammalian sequence, human GPR107 (Genbank:AAK57695.1), identified at an e-value of 3e-63, was aligned to the plant family profile. Schematic above the alignment blocks indicates the 7TM topology of Cand7 as predicted by TMHMM. The triangle denotes the signal peptide cleaveage site, blue lines, extracellular regions, blue blocks, TM domains; red lines, intracellular regions. The “W” indicates the Cand7 Trp193 residue nearly completely conserved across plants and metazoa.


                                                                                                                                            
At5g18520.1    ---------------MAKMPLSVVVFLLFSAAFLAVSMAEIKSLVISDDARPMILFEKFGFTHTGHVTVSISSVSVVST----------------------------------------------
At3g09570.1    ---------------MTRLPL-FVVLLLFSASCITRTAAEIKSLTISDDSRPMILFEKFGFTQSGHVSVSISSVAVVSS----------------------------------------------
At5g02630.1    ---------------MTILPFLAAVFVLQ--LLSTLTVAEIKSFTISNDSRPVILLEKFGIIEIGHVTVSVSSVSVLS-----------------------------------------------
At5g42090.1    ----------------MDLSSRFSSILILLLISISIASAEIRKSEIRSDDRPIIPLDEFGFTHSGRLELDASKIWLSNS----------------------------------------------
Pop554569      ------------------MATRFTLFSLFTSLLLSLSLAEIRFSDIRSDDRQIIPFDEFGFTHFGRLELNVTNIRLSNP----------------------------------------------
Pop797267      ------------------MASRFLLFFLFTSLLLSISLAEIHFSEIRSDDRQIIPFDEFGFTHLGRLELNVTNIHLSNP----------------------------------------------
Os06g04130.1   ------------MAAASPLAAAAALALLLLLLLAAPAAAEIRETVIRSDPRSIIPLDEFGFSHSGVLELNVSGIAFDPP----------------------------------------------
Os02g40550.1   ----MHVRSPARSMDTSLSIAVAAAAVVLLLLVR-GADAEIRTTLIVSDARPLILFEQFGFERGGKATISIRRSFWNLRRG--------------------------------------------
Os04g42960.1   -------------MAASVSLAVAATAVVALLLALPVALAEIKTTPIVADSRPVILFEEFGFKPGGVSAVSVRGVSWRVAEG--------------------------------------------
Pop241510      ---------------------LLILYALFLTSSIPFASSEIKDTYIFDDSRPIIMFEQFGFTEGGQVAISIKDVSWKSR----------------------------------------------
Pop256636      -------------------------------SLLSLSTAEIKTLSITNDARAMILFEKFGFTPTGHVNISVHSVSVASS----------------------------------------------
Pop561523      ---------------MTKPPH-LILRLLVLVLLISLSTAEIKNLTITNDARDMIVFEKFGFTPTGHVTISVNSVSVASS----------------------------------------------
Os01g61970.1   -----------MATAAAAARALLLLIAVAGALLRP-AAAEIKQESFKDDSRASILFEKFGFSRRGFVSIAITGARTSSK----------------------------------------------
Os05g38720.1   -------------MAAAARRLLLLAIAVAAVSLAPRAAAEIRTEAFREDPRPTILFEKFGFSKTGAVRIVITGAGISST----------------------------------------------
Os01g61960.1   -----------------------------------------------------------------------------------------------------------------------------
humanGPR107    MAALAPVGSPASRGPRLAAGLRLLPMLGLLQLLAEPGLGRVHHLALKDDVRHKVHLNTFGFFKDGYMVVNVSSLSLNEPEDKDVTIGFSLDRTKNDGFSSYLDEDVNYCILKKQSVSVTLLILDI
                                                                                                                                            
 

                                                                                                                                          
                                                                                                                                            
At5g18520.1    ------SSDPNPEASRLGFFLLSEESLLQVLLE------IQQNPRF------------CVLDSHYVTHLFTFRDLSPP---------PNSRFNQSYPVTSPNEYSLFFANCVPE-TKVSMAVRTE
At3g09570.1    ------SSDPIPDPSRLGFFLMSEESLLQVVLE------IEQNPNF------------CVLDSNYVLHLFTFHDLSPP---------PGSKYEHLYPVMSPNEYSLFFVNCVPE-TKISMKVRTE
At5g02630.1    ---------PILDSSKLGFFVLSEESLPHVLLE------LQQNFSF------------CVLDSHYILHFFTFVDLSPP---------PRSQFSKSYPITSPNDYSLFFANCVPE-TRVSMKVHTE
At5g42090.1    -------NP-DLDLSKVGFFLCTRDAWVHVIQQ------LEEEEIT------------CALQSDLVKHVFTFNNLKGG---------DKSRFSTVFTENDADQYSLVFANCLQQ-VKISMDVRSA
Pop554569      -------NP-DLDRSKIGFFLCTRDSWLHVINQ------LEDGEIA------------CALQSDLIKPVFTFNDLKK----------DHDSLSKIVTQNDADQYTLVFANCLTS-LKVSMDVKSV
Pop797267      -------NP-DLDRSKIGFFLCTRDSWLHVINQ------LEDGEIT------------CALQSDLIKPVFTFNELVK----------DQTSLSKTLTHNDADQYTLVFANCLTS-LKVSMDVKSA
Os06g04130.1   -------ASSELDLSQLGFFLSTLDAWVHVLRQ------LQDLDVT------------CALQADLVKLAYSFDRLRPPSNPAGVEVARSSSFSTAFPVSEPGQYTLVFANCLGGGLKVSMDVRSA
Os02g40550.1   ------SRRTAVDPSLMGFVLISGTQFPKINNA------SAYAAADPGDNGDDGGGSYCVLTSEYALPVLRLGDVPP-----------GGVTT-TVSIDDPDQYAVVFSNCQDG-VEVTVDVYTE
Os04g42960.1   ------SKLQAADPGLMGFILISNSLFFQINNE------SDYAEAT--------GGAFCPLTSKYVLPLFRLKDIAPD----------GNGKG-SVTIDDDDQYTVLFSSCQDG-VEVTMEVRTE
Pop241510      ------SRKAELNPSSMGFFLARDSSFSTIFTN------DSLQSKD---------ESFCVLSSRYVKLLFNFNDLSMN----------TSAYNGSASIDEADEYSLVFGNCQPE-FEVSMYVHTE
Pop256636      ------VNAPNPVSSRLGFFLLSEESRLQVILE------IQENPNF------------CVLDSHYILSLFTFRDLSPP---------PLSSFNQSYPVTAPNEYLLFFANCAPE-TRVSMSVKTQ
Pop561523      ------LNAGNPLSSRLGFFLLSEESRLQVLLE------IQQSPNF------------CVLDSHFILSLFTFRDLSPP---------PLSSFSQSYPVTAPNEYSLYFANCAPE-TRVSMSVKTE
Os01g61970.1   --------LAKAEPDQFGFFLLSDEALFEAIYEQPPPTDLNPNPEPNPG---------CVLSSPYVKPLFSFADLDGN-----------GNYKKTFPVTQPDEYSLFFANCAPE-TAVTMEVRTD
Os05g38720.1   --------FARPDPKQLGFFLLSDESMFQAIYEAQARRPPERREEVAGGGADEPDVSRCILTSPYVKTLFTFHDLKR------------GHYNKTFPVTHPDEYSLYFANCAPE-SLVTMRVRTE
Os01g61960.1   -----------------------------------------------------------------------------------------------------------------------MDVRVE
humanGPR107    SRSEVRVKSPPEAGTQLPKIIFSRDEKVLGQSQEPNVNPASAGNQTQKTQDGGKSKRSTVDSKAMGEKSFSVHNNGGAVS--------FQFFFNISTDDQEGLYSLYFHKCLGKELPSDKFTFSL
                                                                                                                                            


                                                                                                                                                                                                                                                                                      


At5g18520.1    MYN-KDPN---GSKDYLPAGSTQLPTLYSFFFLCYVAFLGFWSYTCWTN---KQTVHRIHLLMAGLLLIKSLNLICAAEDK--HYVKITGTPHG-WDILFYIFQFIRVVLLFTVIILIGTGWSFL
At3g09570.1    MYN-LDPN---GSKDYLPAGSTRLPGLYFFFSLGYLAFLGLWGYACWVN---KRVVHRIHVLMAALLLMKALNLICAAEDK--HYVKVTGTPHG-WDVLFYIFQFIRVVLLFTVIVLIGTGWSFL
At5g02630.1    IYHDLYPN---GSRDYLLAGSAQLPGLYLVFFLCYLSFLCFWLCFCWNH---KQIVKRIHLLMTALLLVKSLTLICAAVYK--HYVKVTGTAHG-WNIVFYIFQFISVVLLFMVIVLIGNGWSFL
At5g42090.1    MYN-LEGK--KGGRDYLSAGRTVLPKVYFLFSVIYFSLAATWIYVLYKK---RLTVFAIHFFMLGVVVLKALNLLCEAEDK--SYIKKTGTAHG-WDVLFYIFNFLKGITLFTLIVLIGTGWSFL
Pop554569      MYN-LDRG--GKVRDYLSAGKTILPRVYYLLSLIYFGLVGVWIYVLYRK---RLTVYRIHFFMLAVVILKTVNLLCEAEDK--SYIKRTGYAHG-WDVLFYIFSFLKGITLFTLIVLIGTGWSFL
Pop797267      MYN-LDKG--GKARDYLSAGKTMLPRVYYLLSLIYFGLAGVWIYVLYKK---RLTVYRIHFFMLAVVILKTMNLLCEAEDK--SYIKRTGYAHG-WDVLFYIFSFLKGITLFTLIVLIGTGWSFL
Os06g04130.1   MYN-VDPP--TGERSYLSAGATALPTIFGFFGVAYAALAAGWIAILLRK---RAAVFRIHYFMLAVLVLKAVNLLAEAEDK--SYIERTGTAHG-WDVLFYIFSFLKGISLFTLIVLIGTGWSFL
Os02g40550.1   MYN-VRDGISDGPRDYLPVGLRPLPTIYTVVSEVYFAFLALWACVCVRH---RATVERIHAVMGALLLFKALKMACAAEDS--WYVERTGTPHG-WDVAFYVFGFFKGVLLFTVIILIGTGWSIL
Os04g42960.1   MYN-VRPGGGRGVREYLPVGLLPLPGIFAAASAVYFVFLGAWAWACARH---RATAGQIHAVMGALLLFKALKLACAAEDA--WYVERTGTPHG-WDVAFYVFGFFKGVLLFTVIVLIGTGWSFL
Pop241510      MYN-LQDG----AKNFLPIGQTFLPKFFLSMFLIYTCFFGIWSFVCFKQ---RPTVDMIHLIMGALLFVKALKMICASEDE--MYVSKTGTPHG-WDVAFYIFGFFKGIMLFTVIILIGTGWSFL
Pop256636      VYN-LDRD---GSKDYLSAGLTQLPSLFFLYFLAYAAFLGLWIYVCYNN---KRSVHRIHLLMGGLLLMKALNLICAAEDK--HYVKVTGTPHG-WDVLFYIFQFIRVVLLFTVIVLIGTGWSFL
Pop561523      IYN-LDRD---GSRDYLSAGLTQLPSLYSLYFIAYAGFLGLWIYVLYSN---KRSVHRIHLLMGGLLLMKALNLICAAEDK--HYVKVTGTPHG-WDVLFYIFQFIRVVLLFTVIVLIGTGWSFL
Os01g61970.1   MYN-TNLD---GSKDYLSVGQAPVPAIYAFFTVCYLVFLAVWLYVTLYR--NRLSAHRIHHLMSGLLAARMLYCISAAEDQ--HYIRIAGTPHG-WDVMFYLFQLVKGVILFAVIALIGTGWSFL
Os05g38720.1   MYN-GNAD---GSVDYLPVGQAPVPAIYGFFAACYAAFLAAWGYLTLSSRDHRAAAHQIHHLMSGLLAARLLYCLSAAEDQ--HYIRVTGTPHG-WDVAFYLFQLVKGVVLFAVIVLVGTGWSFL
Os01g61960.1   LSD------------SNPDGGEDPVAMIYSFAVCYGVFLIAWLHRTLARG--CSTARPVHDVMSGLLAALMLHCLTAAAHDGRYTSVVAGTARG-WNVPCLALRLVKNAMLFPVVALIGAGWSLP
humanGPR107    DIEITEKN----PDSYLSAGEIPLPKLYISMAFFFFLSGTIWIHILRKR---RNDVFKIHWLMAALPFTKSLSLVFHAIDY--HYISSQGFPIEGWAVVYYITHLLKGALLFITIALIGTGWAFI
 


                                                                                                                                                                                                                                                                                 
                                                                                                                                            
At5g18520.1    KPFLQEKEKNVLIIVIPLQVLANIASIVIGETGPFIKDWVTWNQVFLLVDIICCCAIIFPIVWSIRSLRETSKTDGKAARNLSKLTLFRQFYIVVIGYLYFTRIVVFALKTIA--AYKYQWVSFA
At3g09570.1    KPFLQEKEKNVLMVVVPLQVLANIASIVIGETGPFIKDWVTWNQIFLLVDIVCCCAILFPIVWSIRSLRETSKTDGKAARNLAKLTLFRQFYIVVIGYLYFTRIVVFALKTIA--AYKYRWVSNA
At5g02630.1    KPKLHVKEKKLLVIVVPLQVLANIASIVIGETGPYTQDWVSWNQIFFLADITCCCAIVFAMVWSMCCLRETSKTDGKAVKNLAKLPVLRKFYVLVIGYLFFTRIVVVVMKMKA--DFTYQWVSNA
At5g42090.1    KPYLQDKEKKVLMIVIPLQVVANFAQVVIDETGPYGQDWVTWKQIFLLVDVVCCCAVLFPIVWSIKNLREAAKTDGKAAVNLVKLTLFRQYYIVVICYIYFTRVVVYALETIT--SYKYMWTSVV
Pop554569      KPYLQDKEKKVLMIVIPLQVVANIAQVVIDETGPYGQDWITWKQVFLLVDVVCCCAVLFPIVWSIKNLREAARTDGKAAVNLMKLTLFRQYYIVVICYIYFTRVVVYALETIT--SYKYLWTSVV
Pop797267      KPYLQGKEKKVLMIVIPLQVVANIAQVVIDETGPYGQDWITWKQVFLLVDVVCCCAVLFPIVWSIKNLREAARTDGKAAVNLMKLTLFRQYYIVVICYIYFTRVVVYALETIT--SYKYLWTSVV
Os06g04130.1   KPYLADREKKVLMVVIPLQVVANIAQVVIDESGPYARAWVTWKQVLLLVDVICCCAVLFPIVWSIKNLREAARSDGKAAVNLMKLTLFRQYYVVVICYIYFTRVVVYALMTIT--SYRYQWTSYV
Os02g40550.1   KPYLQEREKNMLMIVIPLQVVENLLLVVIGETGPTGQDWVVWNQVFLLVDVICCCAVFFPIIWSIRSMREASKTDGKAALNLQKLTLFKRFYLVVVGYLYFTRIIASAFLALL--SYKYQWGVNV
Os04g42960.1   KPYLQEREKKVLMIVIPLQVVENIASAVIGETGPAGRDWLAWNQIFLLVDVICCCAVFFPIIWSIRNLREASKTDGKAARNLKKLTLFKQFYLVVVGYLYFTRIAVSAFAAVL--SYRYQWVVTV
Pop241510      KPYLQEREKNVLMIVIPLQVLENIAYVVISETGPATKDWWTWNQIFLLIDVICCCAVFFPIVWSIRNLKEASKSDGKAARNLEKLTLFKNFYICLVMYLYFTRVVVSSMEGIL--DYRYEGFTYV
Pop256636      KPFLQEKEKKVLMVVIPLQVLANIASVVIGETGPFIKDWVTWNQVFLLVDIICCCAIIFPIVWSIRSLRENSKTDGKAARNLAKLQLFRQFYIVVIGYLYFTRIVVFALKTIA--AYKYQWVSNA
Pop561523      KPFLQEKEKKVLMVVIPLQVLANIASVVIGETGPFIKDWVTWNQVFLLVDIICCCAIIFPIVWSIRSLRETSKTDGKAARNLAKLQLFRQFYIVVIGYLYFTRIVVFALKTIA--AYKYQWVSNA
Os01g61970.1   KPFLQDKEKKVLMVVIPLQVAANIAAAVVGETGPFLQGWVTWNQIFLFVDVACCCAVLFPVVWSMRSLRESSKTDGKAARTLAKLTLFRQFYVVVIGYLYFTRIIVYALKTIT--NYKYRWVSVA
Os05g38720.1   RPVLQDREKKVLMVVIPLQVMANIASAVIGETGPFLQGWVTWNQILLFVDVACCCAVLFPVVWSMRSLRETSKTDGKAARTLSKLTLFRQFYIVVIGYLYFTRIVVYALKTIA--SYQFRWVSVL
Os01g61960.1   EPFVQARELNVLTAMVPLQVYMAIATTLSGDGG----VAWTWGHAFVLVQLACCVAVLMPMGRAIRALRKEADTDDKAARRLGKLALFRQLYLAVAVYLYHTWMAVFILKLLVGASSGYRWASVA
humanGPR107    KHILSDKDKKIFMIVIPLQVLANVAYIIIESTEEGTTEYGLWKDSLFLVDLLCCGAILFPVVWSIRHLQEASATDGKAAINLAKLKLFRHYYVLIVCYIYFTRIIAFLLKLAVP--FQWKWLYQL
                                                                                                                                            
  
   


                                                                  


                                                                                      
At5g18520.1    AEEIVSLVFYVIMFHMFRPEEKNEYFAVDD-DEEEAAALALRDE-EFEL----------------------
At3g09570.1    AEEIASLAFYMLMFYMFRPVEKNEYFVIDE-EEEEAAELALKED-DFEL----------------------
At5g02630.1    AEEIATLSFYCLMFYMFRPIEKNEYCDVDD-E-EEIVELSLK-----------------------------
At5g42090.1    ASELATLAFYLFTGYKFRPEVHNPYFVVDD-EEEEAAAEALKLEDEFEL----------------------
Pop554569      AGELATLAFYVFTGYKFKPEAHNPYFVVDD-EEEEAAAEALKLEDEFEL----------------------
Pop797267      AGELATLAFYFFTGYKFKPEAHNPYFVVDD-EEEEAAAEALKLEDEFEL----------------------
Os06g04130.1   AKELATLAFYVFTGYKFRPEVHNPYFAIDD-EEEEAAAEALKLDDEFEL----------------------
Os02g40550.1   AIEAASLAFYLFVFYNFQPVAKNPYLYIGDTVEDAAVEREMDDEGRF------------------------
Os04g42960.1   AMEAASLAFYIFVFYNFKPVENNPYLYVGEDEEEEASG-QLEMEGTFEI----------------------
Pop241510      LSEGASLAFYVFIFYNFQPTERNPYLVIDE-EEELAAEQILQDDDSFEL----------------------
Pop256636      AEETASLLFYVVIFYMFRPVEKNEYFVLDE-EEEEAAELALRDE-EFEL----------------------
Pop561523      AEETASLMFYMVIFYMFRPVEKNEYFVLDE-EDEEAAELALRDE-EFEL----------------------
Os01g61970.1   AEEVATVAFYLFMFYMFRPAERNQYFALDE-DEEEAAELALREE-EFEL----------------------
Os05g38720.1   AEEVATLAFYLFMFYTFRPAERSRYFSFDE-DEEEAAEMVLREE-EFEL----------------------
Os01g61960.1   VDEAAALAFYLFMFCMFSPAEED--IQLEEYTEELIQGGV-------------------------------
humanGPR107    LDETATLVFFVLTGYKFRPASDNPYLQLSQEEEDLEMESVVTTSGVMESMKKVKKVTNGSVEPQGEWEGAV
