# Supplementary material for: Whole proteome identification of plant candidate G-protein coupled receptors in Arabidopsis, rice, and poplar: computational prediction and in-vivo protein coupling
Source: Genome Biol. 2008 Jul 31;9(7):R120. doi: 10.1186/gb-2008-9-7-r120 (PMC2530877; doi:10.1186/gb-2008-9-7-r120)
Supplement: Additional data file 8 — Arabidopsis sequences removed from our analysis by our QFC ion channel filter. [file gb-2008-9-7-r120-S8.doc]

Additional Data File 8. **Two categories of *Arabidopsis* sequences removed from our analysis by our QFC ion channel filter.** The ion channel filter removed a total of 554 non-redundant sequences, including 70 ion channel related sequences and 19 sequences that would have been considered candidate GPCRs based on a direct prediction as a GPCR by the native QFC analysis and at least a “2/3” prediction as a 7TM protein. Loci in boldface are members of the greater Cand6/7 superfamily (see Additional data file 4 for more detail).

| **T7Locus** | | **ID** | **Description** |  |
| --- | --- | --- | --- | --- |
| Channel related | |  |  |  |
| At1g02510.1 | KCO4, TPK4 | | KCO4 (Ca2+ activated outward rectifying K+ channel 4) | |
| At1g05300.2 | ZIP5 | | ZIP5 (ZINC TRANSPORTER 5 PRECURSOR); cation transporter | |
| At1g05580.1 | CHX23 | | ATCHX23 (CATION/H+ EXCHANGER 23); monovalent cation:proton antiporter | |
| At1g05580.2 | CHX23 | | ATCHX23 (CATION/H+ EXCHANGER 23); monovalent cation:proton antiporter | |
| At1g06470.1 |  | | phosphate translocator-related | |
| At1g08135.1 | CHX6B | | ATCHX6B/CHX6B (CATION/H+ EXCHANGER 6B); monovalent cation:proton antiporter | |
| At1g08140.1 | CHX6A | | ATCHX6a (CATION/H+ EXCHANGER 6A); monovalent cation:proton antiporter | |
| At1g09860.1 | PUP16 | | ATPUP16 (Arabidopsis thaliana purine permease 16); purine transporter | |
| At1g12480.1 |  | | C4-dicarboxylate transporter/malic acid transport family protein | |
| At1g12600.1 |  | | similar to ATUTR2/UTR2 (UDP-GALACTOSE TRANSPORTER 2) | |
| At1g14660.1 | NHX8 | | ATNHX8 (Arabidopsis thaliana Na+/H+ exchanger 8); sodium:hydrogen antiporter | |
| At1g27080.1 |  | | proton-dependent oligopeptide transport (POT) family protein | |
| At1g31770.1 |  | | ABC transporter family protein | |
| At1g32450.1 |  | | proton-dependent oligopeptide transport (POT) family protein | |
| At1g47240.1 | NRAMP2 | | NRAMP2 (NRAMP metal ion transporter 2); metal ion transporter | |
| At1g53660.1 |  | | phosphate translocator-related | |
| At1g54370.1 | NHX5 | | ATNHX5/NHX5 (NA+/H+ ANTIPORTER 5); sodium:hydrogen antiporter | |
| At1g57943.1 | PUP17 | | ATPUP17 (Arabidopsis thaliana purine permease 17) | |
| At1g57943.2 | PUP17 | | ATPUP17 (Arabidopsis thaliana purine permease 17) | |
| At1g60160.1 |  | | potassium transporter family protein | |
| At1g64170.1 | CHX16 | | ATCHX16 (CATION/H+ EXCHANGER 16); monovalent cation:proton antiporter | |
| At1g69870.1 |  | | proton-dependent oligopeptide transport (POT) family protein | |
| At1g79610.1 |  | | sodium proton exchanger, putative (NHX6) | |
| At2g01110.1 | UNE3, APG2 | | APG2 (ALBINO AND PALE GREEN 2) | |
| At2g04620.1 |  | | cation efflux family protein | |
| At2g13620.1 | CHX15 | | ATCHX15 (cation/hydrogen exchanger 15); monovalent cation:proton antiporter | |
| At2g20780.1 |  | | mannitol transporter, putative | |
| At2g27240.1 |  | | similar to putative aluminum activated malate transporter [B. napus] (GB:BAE97280.1) | |
| At2g28900.1 | OEP16-1 | | OEP16 (OUTER ENVELOPE PROTEIN 16); protein translocase | |
| At2g41190.1 |  | | amino acid transporter family protein | |
| At2g42210.1 | OEP16-3 | | ATOEP16-3; protein translocase | |
| At2g42210.2 | OEP16-3 | | ATOEP16-3; protein translocase | |
| At2g46440.1 | CNGC11 | | ATCNGC11 (cyclic nucleotide gated channel 11); cyclic nucleotide binding / ion channel | |
| At2g46450.1 | CNGC12 | | ATCNGC12 (cyclic nucleotide gated channel 12); cyclic nucleotide binding / ion channel | |
| At3g01350.1 |  | | proton-dependent oligopeptide transport (POT) family protein | |
| At3g04800.1 | TIM23-3 | | ATTIM23-3 (A. thaliana translocase inner membrane subunit 23-3); protein translocase | |
| At3g06450.1 |  | | anion exchange family protein | |
| At3g12100.1 |  | | cation efflux family protein / metal tolerance protein, putative | |
| At3g12100.2 |  | | cation transporter/ efflux permease | |
| At3g19553.1 |  | | amino acid permease family protein | |
| At3g21670.1 | NTP3 | | nitrate transporter (NTP3) | |
| At3g44910.1 | CHX12 | | ATCHX12 (cation/H+ exchanger 12); monovalent cation:proton antiporter | |
| At3g44920.1 | CHX11 | | ATCHX11 (cation/H+ exchanger 11); monovalent cation:proton antiporter | |
| At3g44930.1 | CHX10 | | ATCHX10 (CATION/H+ EXCHANGER 10); monovalent cation:proton antiporter | |
| At3g52080.1 | CHX28 | | CHX28 (cation/hydrogen exchanger 28); monovalent cation:proton antiporter | |
| At3g52310.1 |  | | ABC transporter family protein | |
| At3g58810.1 | MTPA2 | | MTPA2; efflux permease/ zinc ion transporter | |
| At3g61940.1 | MTPA1 | | MTPA1; efflux permease/ zinc ion transporter | |
| At4g03560.1 | TPC1, FOU2 | | ATTPC1 (TWO-PORE CHANNEL 1); calcium channel/ voltage-gated calcium channel | |
| At4g04850.1 | KEA3 | | KEA3 (K+ efflux antiporter 3); potassium:hydrogen antiporter | |
| At4g13420.1 | HAK5 | | HAK5 (High affinity K+ transporter 5); potassium ion transporter | |
| At4g17550.1 |  | | transporter-related | |
| At4g18190.1 | PUP6 | | ATPUP6 (Arabidopsis thaliana purine permease 6); purine transporter | |
| At4g18790.1 | NRAMP5 | | NRAMP5 (NRAMP metal ion transporter 5); metal ion transporter | |
| At4g21680.1 |  | | proton-dependent oligopeptide transport (POT) family protein | |
| At4g23700.1 | CHX17 | | ATCHX17 (CATION/H+ EXCHANGER 17); monovalent cation:proton antiporter | |
| At4g32510.1 |  | | anion exchanger | |
| At4g33530.1 | KUP5 | | KUP5 (K+ uptake permease 5); potassium ion transporter | |
| At4g38050.1 |  | | permease | |
| At5g01680.1 | CHX26 | | ATCHX26 (cation/hydrogen exchanger 26); monovalent cation:proton antiporter | |
| At5g01690.1 | CHX27 | | ATCHX27 (cation/hydrogen exchanger 27); monovalent cation:proton antiporter | |
| At5g09400.1 | KUP7 | | KUP7 (K+ uptake permease 7); potassium ion transporter | |
| At5g19600.1 | SULTR3;5 | | SULTR3;5 (SULTR3;5); sulfate transporter | |
| At5g19640.1 |  | | proton-dependent oligopeptide transport (POT) family protein | |
| At5g25430.1 |  | | anion exchange protein family | |
| At5g40890.1 | CLC-A | | ATCLC-A (CHLORIDE CHANNEL A); anion channel/ voltage-gated chloride channel | |
| At5g41610.1 | CHX18 | | ATCHX18 (cation/hydrogen exchanger 18); monovalent cation:proton antiporter | |
| At5g46050.1 | PTR3 | | ATPTR3/PTR3 (PEPTIDE TRANSPORTER PROTEIN 3); transporter | |
| At5g51710.1 | KEA5 | | KEA5 (K+ efflux antiporter 5); potassium:hydrogen antiporter | |
| At5g55630.1 | KCO1, TPK1 | | KCO1 (CA2+ ACTIVATED OUTWARD RECTIFYING K+ CHANNEL 1) | |
| Potential Candidates |  | |  | |
| **AT1G10980.1** |  | | similar to unknown protein [Arabidopsis thaliana] (AT1G61670.1); sim to Lung 7TMR | |
| AT1G11000.1 | MLO4 | | MLO4 (MILDEW RESISTANCE LOCUS O 4); calmodulin binding | |
| AT1G11310.1 | PMR2, MLO2 | | MLO2 (MILDEW RESISTANCE LOCUS O 2); calmodulin binding | |
| AT1G12600.1 |  | | similar to ATUTR2/UTR2 (UDP-GALACTOSE TRANSPORTER 2) [Arabidopsis thaliana] | |
| AT1G26700.1 | MLO14 | | MLO14 (MILDEW RESISTANCE LOCUS O 14); calmodulin binding | |
| **AT1G61670.1** |  | | similar to unknown protein [Arabidopsis thaliana] (AT1G10980.1); sim to Lung 7TMR | |
| **AT1G72480.1** |  | | similar to unknown protein [Arabidopsis thaliana] (AT2G01070.1); sim to Lung 7TMR | |
| **AT2G01070.1** |  | | similar to unknown protein [Arabidopsis thaliana] (AT1G72480.1); sim to Lung 7TMR | |
| AT2G17480.1 | MLO8 | | MLO8 (MILDEW RESISTANCE LOCUS O 8); calmodulin binding | |
| AT2G32530.1 | CSLB3 | | ATCSLB03 (Cellulose synthase-like B3) | |
| AT2G32540.1 | CSLB4 | | ATCSLB04 (Cellulose synthase-like B4) | |
| AT2G41050.1 |  | | PQ-loop repeat family protein / transmembrane family protein | |
| AT3G09570.1 |  | | similar to unknown protein [Arabidopsis thaliana] (AT5G18520.1); sim to Lung 7TMR | |
| AT3G45290.1 | MLO3 | | MLO3 (MILDEW RESISTANCE LOCUS O 3); calmodulin binding | |
| AT3G58810.1 | MTP3,MTPA2 | | MTPA2; efflux permease/ zinc ion transporter | |
| AT3G59310.1 |  | | similar to unknown protein [Arabidopsis thaliana] (AT3G59340.1); | |
| AT4G20100.1 |  | | PQ-loop repeat family protein / transmembrane family protein | |
| AT5G42090.1 |  | | similar to unknown protein [Arabidopsis thaliana] (AT3G09570.1); sim to Lung 7TMR | |
| AT5G53760.1 | MLO11 | | MLO11 (MILDEW RESISTANCE LOCUS O 11); calmodulin binding | |
