# Supplementary material for: Whole proteome identification of plant candidate G-protein coupled receptors in Arabidopsis, rice, and poplar: computational prediction and in-vivo protein coupling
Source: Genome Biol. 2008 Jul 31;9(7):R120. doi: 10.1186/gb-2008-9-7-r120 (PMC2530877; doi:10.1186/gb-2008-9-7-r120)
Supplement: Additional data file 10 — Bioinformatic characterization of the Arabidopsis proteome (version 6) sequences predicted to be candidate GPCRs by PRED-GPCR. [file gb-2008-9-7-r120-S10.doc]

Additional Data File 10. **Characterization of the candidate GPCRs predicted by PRED-GPCR in the Arabidopsis proteome (v6).** The setting column indicates the PRED-GPCR settings used during the whole proteome analyses. Only a single sequence was predicted as a GPCR by the QFC (indicated by a “yes”) and none were predicted as a GPCR by GPCHMM. The predicted number of transmembrane domains and the intracellular (in) or extracellular (out) localization of the N-terminus are shown. Pcut-T and Pcut-H describe topology predictions of the mature proteins by TMHMM and HMMTOP, respectively, after in-silico cleavage at the signal peptide cleavage site predicted by Phobius.

| **Locus** | **Setting** | **QFC** | **GPCRHMM** | **TMHMM** | **HMMTOP** | **Phobius** | **Pcut-T** | **Pcut-H** | |
| --- | --- | --- | --- | --- | --- | --- | --- | --- | --- |
| At1g29570.1 | Default | - | - | 0 | 0 | 0 |  | |  |
| At1g52780.1 | Default | - | - | 6 (in) | 8 (in) | 7 (out) | 5 (out) | | 8 (in) |
| At1g55350.1 | Default | - | - | 23 (in) | 24 (out) | 23 (out) |  | |  |
| At1g58848.1 | Default | - | - | 0 | 2 (out) | 2 (out) |  | |  |
| At1g61300.1 | Default | - | - | 0 | 0 | 0 |  | |  |
| At3g28770.1 | Default | - | - | 1 (in) | 1 (out) | 0 | 0 | | 0 |
| At4g35940.1 | Default | - | - | 0 | 0 | 0 |  | |  |
| At4g19050.1 | Default/User | - | - | 0 | 1 (in) | 0 |  | |  |
| At1g72840.1 | User | - | - | 0 | 0 | 0 |  | |  |
| At2g36630.1 | User | yes | - | 9 (in) | 11 (out) | 11 (out) |  | |  |
| At2g27630.1 | User | - | - | 0 | 0 | 0 |  | |  |
| At3g43240.1 | User | - | - | 0 | 0 | 0 |  | |  |
| At3g60380.1 | User | - | - | 2 (out) | 2 (in) | 2 (in) |  | |  |
| At4g27190.1 | User | - | - | 0 | 1 (in) | 0 |  | |  |
| At4g27220.1 | User | - | - | 0 | 3 (in) | 0 |  | |  |
| At5g24350.1 | User | - | - | 0 | 2 (out) | 1 |  | |  |
| At5g38850.1 | User | - | - | 0 | 0 | 0 |  | |  |
| At5g40100.1 | User | - | - | 0 | 0 | 0 |  | |  |
| At5g57120.1 | User | - | - | 0 | 0 | 0 |  | |  |
