# Supplementary material for: Whole proteome identification of plant candidate G-protein coupled receptors in Arabidopsis, rice, and poplar: computational prediction and in-vivo protein coupling
Source: Genome Biol. 2008 Jul 31;9(7):R120. doi: 10.1186/gb-2008-9-7-r120 (PMC2530877; doi:10.1186/gb-2008-9-7-r120)
Supplement: Additional data file 11 — Venn diagram detailing the extent of overlap between candidate GPCRs predicted by our analysis and that of Moriyama et al. [49]. [file gb-2008-9-7-r120-S11.ppt]

## Slide 1
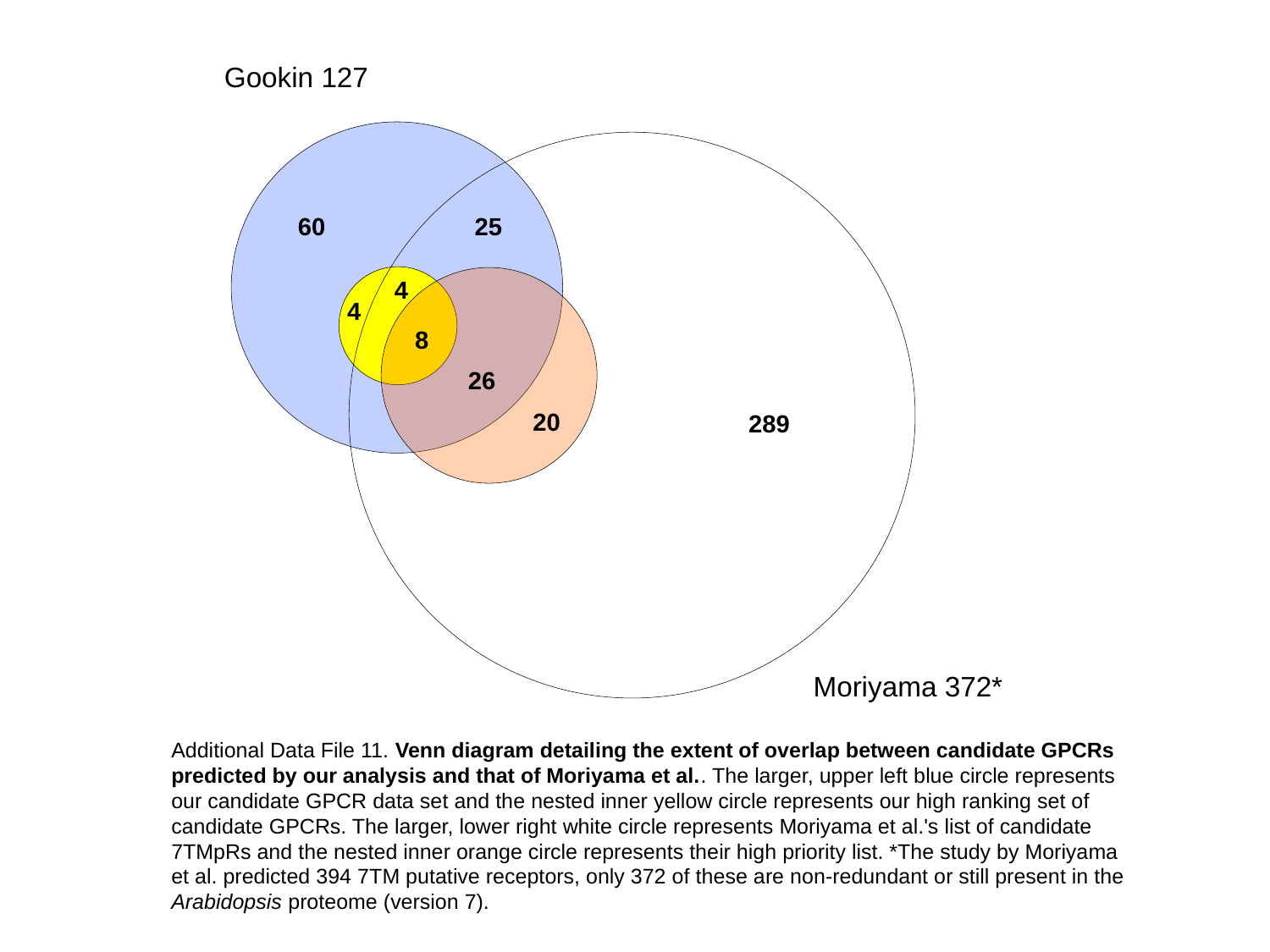

Gookin 127
60
25
4
4
8
26
20
289
Moriyama 372*
Additional Data File 11. Venn diagram detailing the extent of overlap between candidate GPCRs predicted by our analysis and that of Moriyama et al.. The larger, upper left blue circle represents our candidate GPCR data set and the nested inner yellow circle represents our high ranking set of candidate GPCRs. The larger, lower right white circle represents Moriyama et al.'s list of candidate 7TMpRs and the nested inner orange circle represents their high priority list. *The study by Moriyama et al. predicted 394 7TM putative receptors, only 372 of these are non-redundant or still present in the Arabidopsis proteome (version 7).
